# Deciphering the Estrogenic Activity of Aqueous Leachates from Elastomers by Effect-Directed Analysis

**DOI:** 10.1021/acs.est.5c05987

**Published:** 2025-09-30

**Authors:** Rebecca Süßmuth, Timothy Rosenberger, Peter Schweyen, Georg Dierkes, Anna Maria Bell, Arne Wick, Sebastian Buchinger, Thomas A. Ternes

**Affiliations:** Federal Institute of Hydrology, Am Mainzer Tor 1, 56068 Koblenz, Germany

**Keywords:** effect-directed analysis, nontarget screening, (planar) yeast estrogen screen, rubber elastomers, rubber-related additives, antioxidants, 4-hydroxydiphenylamine

## Abstract

This study successfully used effect-directed analysis to identify a transformation product of *N*,*N*′-substituted *p*-phenylenediamines (PPD) rubber-antiozonants as the main driver of estrogenicity in aqueous leachates from elastomer membranes. First, two signals with estrogenic activity were detected via high-performance thin-layer chromatography (HPTLC) in a planar yeast estrogen screen (p-YES). After the active fractions were extracted from the HPTLC plate, they were analyzed by nontarget screening using liquid chromatography coupled to high-resolution mass-spectrometry. Via a prioritization process, the elucidation of the MS^2^-spectra, and a library research, 4-hydroxydiphenylamine (4HDPA) and *N*-phenyl-*p*-benzoquinone monoimine (QMI) were identified (*m*/*z* 186.0915 and 184.0757, respectively, in positive mode). 4HDPA is a hydrolysis product of PPD antiozonants, whereas QMI is formed by the oxidation of 4HDPA. By measuring the leachate within the YES medium, we found that QMI was transformed into 97 ± 1% of the estrogenic active compound 4HDPA. Therefore, YES results of redox couple compounds need to be carefully assessed due to potential transformations occurring in the YES medium itself. Finally, the estrogenic activity of 4HDPA was identified for the first time, and we confirmed that the overall estrogenicity observed in the elastomer leachates was predominantly caused by the elution and formation of 4HDPA by 120 ± 30%. Furthermore, 4HDPA was found in river and stream water at concentrations ranging between 7 and 20 ng/L.

## Graphical abstract

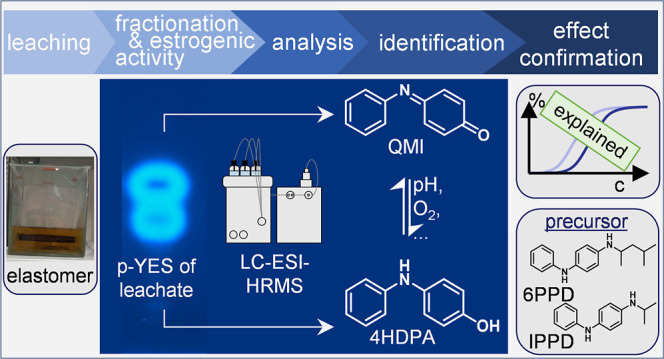

## 1 Introduction

Rubber materials are widely used in technical products and can be found in tires,^1^ seals,^2,3^ plumbing materials for drinking water supply,^4^ hydraulic constructions such as rubber dams,^5^ and recycled as crumb rubbers in sport fields.^6,7^ Elastomer membranes, particularly used for rubber dams, often consist of a blend based on ethylene propylene diene rubber (EPDM) and styrene butadiene rubber for optimized properties, with a layer of polyethylene terephthalate (PET) and polyamide (PA) fabric as reinforcement material, which is embedded into the membrane.^5^ For different product properties of elastomers, a variety of additives are added,^6,8–10^ such as antidegradants and vulcanization accelerators. Especially antioxidants are an important group of additives for the durability of rubber materials. Most often *N*,*N*′-substituted *p*-phenylenediamines (PPD)^11,12^ are used for this purpose and came into focus after the identification of the ichthyotoxic 6PPD-quinone, which is a transformation product (TP) of *N*-(1,3-dimethylbutyl)-*N*′-phenyl-*p*-phenylenediamine (6PPD). 6PPD-quinone is toxic in environmentally relevant concentrations to salmonids (coho salmon *Oncorhynchus kisutch*, 24 h LC_50_ of 0.095 μg/L, brook trout *Salvelinus fontinalis*, 24 h LC_50_ of 0.59 μg/L, rainbow trout *Oncorhynchus mykiss*, 72 h LC_50_ of 1.0 μg/L).^12–15^ For this reason, there has been growing research on 6PPD-quinone, but information about the toxicity of other TPs is rare.

The leachability of organic compounds from elastomers was already investigated in different studies.^10,16–18^ Heavy metals such as Zn, Cr, and Pb^9,11^ also potentially leach from such materials. Via direct contact with water, e.g., in hydraulic constructions^19^ or indirectly via runoff and leaching of, e.g., tire particles after a precipitation event,^17,20,21^ heavy metals, organic compounds, and their related TPs can be released into the aquatic environment^16,22–26^ and potentially cause toxic effects. Due to the scarcity of toxicological information, particularly in the case of TPs, potential environmental risks of compounds released from elastomers are often unknown. A recent study reported different toxic effects caused by tire-related chemicals.^18^ A number of further studies examined the estrogenicity of tire leachates using an enzyme-linked reporter assay (ELRA)^27^ and in tire extracts using the ERα-CALUX.^28^ In both studies, substances causing the observed estrogenicity have not yet been identified. It was hypothesized that detected polycyclic aromatic compounds could contribute to the observed effect.^28^

Anyway, estrogenic active substances released into the environment contribute to an existing xenoestrogenic pollution and might lead to intersex in different species^29,30^ and, therefore, affect whole populations by a shift in the sex ratio.^29,31^ For this reason, endocrine substances became a subject of regulation and were listed in the annex VIII of the EU Water Framework Directive.^32^ Therefore, the aim of the present study was to identify the main drivers of previously detected estrogenicity in leachates of an elastomer membrane by effect-directed analysis (EDA). EDA is applied to identify substances causing specific toxic effects in complex samples, such as leachates, which might contain thousands of chemicals.^33,34^ Toxic drivers may be identified by EDA through biotesting, sequential and different separation techniques, and chemical analysis of fractions exhibiting specific toxic effects.^35,36^ Previous studies have shown that EDA can be complemented by planar bioassays performed directly on plates after sample separation by high-performance thin-layer chromatography (HPTLC).^22,37,38^ Substances could be detected that are responsible for specific effects such as estrogenic, antiandrogenic, and genotoxic effects.^22,36^ One example for this approach is the planar yeast estrogen screen (p-YES), a commonly known bioassay that is currently under standardization (DIN/TS 38415-10),^38–43^ which can easily be performed for the identification of estrogenic effects.^39^ By extraction of estrogenic active fractions from the HPTLC plate and comparison of released features, relevant candidates were prioritized. Finally, the responsible substance could be determined and confirmed by determining its estrogenic potential.

## 2 Methods and Materials

### 2.1 Concept of the EDA Approach

In the following section, an overview of the EDA approach is illustrated in Figure 1. For sample preparation, the elastomer membrane was leached in ultrapure water and enriched by solid phase extraction (SPE).

**Figure 1:**
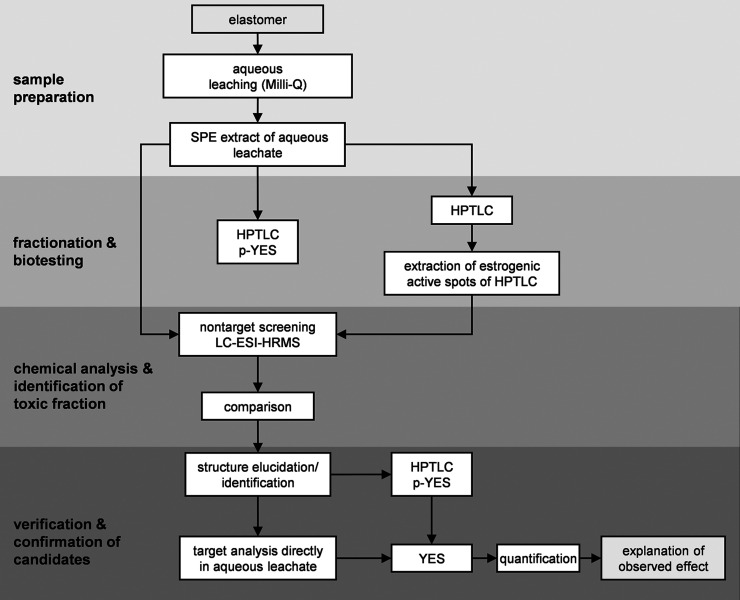
General approach of our EDA study modified after Brack et al.^35^ The leachates were prepared by using ultrapure water (Milli-Q) and enriched by solid phase extraction (SPE) for fractionation with high-performance thin-layer chromatography (HPTLC). Estrogenicity was determined by the planar and nonplanar yeast estrogen screens (p-YES/YES). Liquid chromatography electrospray ionization high-resolution mass spectrometry (LC-ESI-HRMS) was performed for nontarget screening (NTS)

Subsequently, the extracts were analyzed with the p-YES to identify estrogenic active fractions on the HPTLC plate. Furthermore, the active fractions were extracted from a separately developed HPTLC plate. After identification of the toxic fractions, chemical analysis was performed via nontarget screening (NTS) using liquid chromatography coupled to high-resolution mass spectrometry (LC-HRMS) with SPE extracts and the extracted HPTLC fractions. Two possible candidates were tentatively identified by elucidation of the MS^2^-spectra as well as using an in-house database and a literature search. For verification and confirmation, the chemical structures were confirmed by measuring authentic standards. Their estrogenic activity was screened with the p-YES. The two identified substances were further examined with the nonplanar YES, and one substance was quantified in the nontreated leachate (without SPE enrichment). The contribution of the identified compound on the overall estrogenicity of the leachate was calculated using its concentration in the leachate with regard to the 17β-estradiol equivalents (E2-EQ) determined with the YES.^44,45^

### 2.2 Chemicals

Information on all used chemicals and solvents is provided in Table S1 in the Supporting Information. In addition, *N*-phenyl-*p*-benzoquinone monoimine (QMI, 2406-04-4) was synthesized by oxidation of 4-hydroxydiphenylamine (4HDPA) using silver carbonate on Celite in toluene^46–48^ (details and nuclear magnetic resonance (NMR) supplied in Supporting Information, Figure S1 and S2) and confirmed by ^13^C NMR using the Spinsolve 80^ULTRA^ (Magritek, Aachen, Germany). Ultrapure Water (Milli-Q) was obtained by a Milli-Q IQ 7003 water purification system (Merck, Darmstadt, Germany).

### 2.3 Sample Preparation

An elastomer membrane was analyzed, which is of similar composition to the membrane used for the rubber gates of the weir at the Nordumfluter (Section 2.7). It consists of EPDM with a styrene component (details supplied in the Supporting Information, Figures S3 and S4). The aqueous leachates were obtained by exposing the material to Milli-Q in a glass tank (300 × 220 × 240 mm) in triplicate according to Bell et al.^39^ and Rosenberger et al.^49^ The tank was covered with a glass plate, and the material was fixed with nylon strings. The ratio of water volume to surface was adjusted to 33 L/m^2^. After 7 days, a part of the leachate was stored in an amber glass bottle at −20 °C. Milli-Q was used for leaching due to less interfering substances, which improves the conditions for an identification by EDA. Another 1000 mL of the aqueous leachate was concentrated 500× by SPE using an OASIS HLB 6 cm^3^ (200 mg) (further described as “extract”) and stored in an amber glass bottle at −20 °C as well. The cartridge was preconditioned using 2 mL of *n*-heptane, 2 mL of acetone, 3 × 2 mL of methanol, and 4 × 2 mL of double distilled water. After loading, the cartridges were dried and eluted with 4 × 2 mL of methanol, and the sample volume was reduced by evaporation. They were filled up with methanol to a final volume of 2 mL. For chemical analysis, the extracts were diluted to 1× and 5× relative enrichment factor (REF) (1×-extract and 5×-extract).

### 2.4 Bioassays

For bioassays, the extract was diluted with methanol to the used REF. A yeast strain, according to McDonnell et al.,^50,51^ was used for testing. The yeast strain contains a reporter gene (lacZ) under the control of an estrogen-responsive element (ERE). After exposure to samples, estrogenicity can be visualized by the conversion of a substrate into a visually detectable product by the induced enzyme β-galactosidase.

#### 2.4.1 p-YES

The p-YES^42,52^ was performed for the visual detection and identification of estrogenic compounds. For HPTLC, MERCK silica gel 60 F254 (20 × 10 cm) plates were used. The plates were prewashed using 5 mL of methanol to 5 mm just below the upper edge, activated at 110 °C for 30 min,^42^ and stored wrapped in aluminum foil inside a desiccator. The samples were applied at 8 mm plate-height in bands of 5 mm width with a CAMAG Automatic TLC Sampler (ATS 4). Development was done with a CAMAG Automatic Development Chamber (ADC 2) up to 70 mm by using a low polarity developing solvent (9:1, toluene/ethyl acetate).^53^ The chamber was saturated for 20 min using a saturation pad. Relative humidity was adjusted to 33% for 10 min with a saturated magnesium chloride solution. Thereafter, 3 mL of the test culture^50,51^ was sprayed on the developed plates with a CAMAG Derivatizer (yellow nozzle, spray intensity 5), and plates were incubated for 3 h at 30 °C and 90% relative humidity in plastic boxes (NuAire CO_2_-incubator NU-5820E). Subsequently, the plates were sprayed with 2.5 mL of 4-methylumbelliferyl-β-d-galactopyranoside (MUG) solution (green nozzle, spray intensity 5) and incubated for 15 min at 37 °C (Memmert IPP 400). Images of the applied samples and the developed plates were taken with a CAMAG Visualizer 2 at white light, 254 and 366 nm, and evaluated using an adapted version of the “extrapolation-cytotoxic-masking” script for the calculation of the retention factor (*R*_f_-)­values.^49,54^ The positive control (PC) consisted of a mixture of four different estrogens: estrone (E1; 1 ng/mL), 17α-ethinylestradiol (EE2; 0.1 ng/mL), 17β-estradiol (E2; 0.1 ng/mL), and estriol (E3; 10 ng/mL).

For identification of suspected substances in the p-YES, estrogenic active fractions (corresponding to p-YES signals) were extracted from a separately developed HPTLC plate. For the second HPTLC plate, 45 μL 100× REF extract was applied as a 75 mm wide band on one-half of the plate, and 45 μL of methanol (process blank) was applied on the other half. The plate was developed under similar conditions as described before. After development, the *R*_f_ of the signals was determined, and plates were cut horizontally between the two fractions of interest at a height of 30.5 mm and vertically between the two applications at a width of 100 mm with a CAMAG smartCut device (Figure S6). Subsequently, the fractions were scratched from the surface with a scalpel and transferred to 15 mL centrifuge tubes prefilled with 2.5 mL of methanol, agitated for 3 min, and afterward centrifuged at 2700 RCF for 10 min at 20 °C. The supernatants were collected with a syringe and cannula, filtered with a 45 μm PTFE syringe filter to clear the samples from remaining silica, transferred to glass vials, and analyzed using the described NTS approach in Section 2.5.1. For effect verification, the extracted fractions were tested again in the p-YES (Figure S7).

#### 2.4.2 YES

The YES was performed in Greiner Bio-One Cellstar plates according to ISO 19040-1 ^55^ to achieve toxicological parameters for the quantification of the estrogenic potential^56^ of identified compounds (Section 2.6). The tests were incubated for 18 h at 30 °C. A minimum of three independent replicates were performed for each of the samples. Samples were sequentially diluted at 1:2 up to seven dilution levels. E2 served as on-plate PC in a concentration range from 0.66 to 500 ng/L and 1% ethanol was used as a negative control.

### 2.5 Chemical Analysis

#### 2.5.1 NTS

Prior to use, all glassware was heated in an oven at 500 °C. Chemical analysis of the 1× and 5×-extracts was performed using an infinity 1260 LC-system (Agilent, Waldbronn, Germany) connected to a TripleToF 6600 hybrid quadrupole time-of-flight mass spectrometer (QToFMS/MS) (Sciex, Darmstadt, Germany). Chromatographic separation was achieved on a Zorbax Eclipse Plus C18 column (2.1 mm × 150 mm, 3.5 μm, Agilent) with a precolumn (3.0 mm × 4.00 mm, AQ C18, Phenomenex, Aschaffenburg, Germany) at a flow of 300 μL/min and 40 °C using a gradient elution with Milli-Q and acetonitrile, both with 0.1% formic acid (Table S2). For quality assurance, a mix of three internal standards (IS) was added to each sample: bezafibrate-*d*_4_ (*c* = 1.0 μg/L), iopromide-*d*_3_ (*c* = 2.0 μg/L), and olmesartan acid-*d*_6_ (*c* = 0.5 μg/L). The injection volume was 100 μL. Full scan mass spectrum (MS) was obtained via electrospray ionization source (IonDrive) in positive and negative mode (ESI+ and ESI−) in the mass range of 100 to 1200 Da. Details on the NTS approach can be found in Köppe et al.^57^ The obtained wiff-files were converted into the open data format mzXML with Proteowizzard (3.0), and peak picking of the measured data was performed using an algorithm written in R.^58^ Settings used were: minimum intensity (counts) of 10, signal-to-noise (S/N) ratio of 3, and peak width (s) 5 to 60.^57,59^ Library search was done with an in-house collective spectral library (CSL)^59^ with an allowed difference of 1 min in retention time (RT) and MS^1^ tolerance of 10 mDa and 15 mDa for MS^2^ tolerance. Identification of features detected in the NTS was performed according to Schymanski et al.:^60^ a confirmation level (CL) of 4 corresponds to the unequivocal molecular formula (MS isotope/adduct), CL3 to tentative candidates (MS, MS^2^, and experimental data), CL2 to the probable structure (MS, MS^2^, and library search), and CL1 to the confirmed structure (MS, MS^2^, RT, and reference standard).

#### 2.5.2 Standard Addition Approach

Quantification in the leachates was achieved in triplicate by standard addition according to DIN 32633 ^61^ with spiked concentration levels of 210, 420, 630, and 840 μg/L simulating the conditions in the YES as described below by using the incubation medium to compensate matrix effects. Therefore, 20 μL of the leachate was diluted with 60 μL Milli-Q and 40 μL of the McDonnell medium, according to ISO 19040-1,^55^ was added to a final volume of 120 μL. For analysis, the NTS approach was used as described above in Section 2.5.1. The injection volume was 1 μL. Limit of detection (LOD) and limit of quantification (LOQ) were defined with a S/N ratio of 3 and 10, respectively, and since the leachates already contained the analyte, the LOD and LOQ were estimated by extrapolating the measured concentrations to the one corresponding to a S/N ratio of 3 and 10 resulting in a LOD of 0.07 μg/L and a LOQ of 0.23 μg/L. Data evaluation was performed using MultiQuant 3.0 (Sciex, Darmstadt, Germany) and Origin 2024 (Massachusetts, USA). More details can be found in Table S3. Errors were calculated using the 95%-confidence intervals.

#### 2.5.3 pH-Dependent Formation of QMI

A pH-dependent formation of QMI by oxidation of 4HDPA was tested via a full scan using the NTS approach at 4 °C in the autosampler of the LC. 4HDPA was spiked (*c* = 50 μg/L) directly into an LC-vial in triplicate with the respective buffered pH (5–9). 4HDPA and QMI were measured at definite time points (0, 3, 5, 7, 9, 12, 14, 17, 48, 73, 100, 123, 146, and 173 h). The buffered solutions were prepared using the following substances (*c* = 10 mM): for pH 5 acetate, pH 6 bis-TRIS, pH 7 and 8 phosphate, and pH 9 borate buffer (details, Table S1). The injection volume was 10 μL. Data was evaluated using MultiQuant 3.0 (Sciex, Darmstadt, Germany).

### 2.6 Contribution to the Total Estrogenicity

The concentration–response relationship of the E2 PC and the 17β-estradiol equivalent (E2-EQ_sample_) of the extracted leachate was calculated as described in ISO 23196 ^56^ with the use of R (version 4.2.2) and the drc-package (version 3.0.1).^62,63^ Based on these parameters, the relative potency (REP) of the suspected analyte was calculated by dividing the effect concentration (EC_10_) of the respective on-plate E2 PC by the EC_10_ of the analyte (1).^44^ The derived REP was then multiplied with the measured analyte’s concentration in the leachate to calculate its E2-EQ_analyte_ (2). The effect contribution of an identified compound to the overall observed estrogenicity is then given according to eq 3,^45^ by the ratio of the calculated E2-EQ_analyte_ to the overall estrogenic activity of the sample (E2-EQ_sample_). The error of the derived percentage was calculated on the basis of the 95% confidence intervals using Gaussian error propagation by the partial derivation of the individual values.

1 $$REP=\frac{EC_{10,E2}}{EC_{10,analyte}}$$

2 $$E2‐EQ_{analyte}=REP\times C$$

3 $$percentage\;of\;estrogenicity=\frac{E2‐EQ_{analyte}}{E2‐EQ_{sample}}\times 100$$

### 2.7 Occurrence and Semiquantification of 4HDPA in German Surface Waters

The occurrence of 4HDPA and QMI was screened using the NTS-portal.^64,65^ In this database, German federal states can provide their NTS data, which was collected in water and sediment/suspended matter. The samples for this database were analyzed similar to the NTS approach used in our study, including the same IS. In addition, water samples from a monitoring campaign (2024) at the artificial bypass of the river “Spree” (Brandenburg, Germany), named “Nordumfluter” and a movable weir with water-filled rubber gates planed and supplied by Hydroconstruct Ges.m.b.H. (Steyr, Austria) in the canal were screened in retrospective. The length of 25 km extends from the south of the village “Schmogrow” to the town “Lübben”.^66,67^ Mean annual water discharge amounts to 4074 L/s^68^ with a catchment area of 2.81 km^2^.^66^ The weir consists of two fields with a width of 9 m and a height of 1.9 m. The samples were taken upstream and downstream and from inside the membrane of one of the rubber gates and analyzed via the NTS approach. 4HDPA was semiquantified in retrospective. As a rough estimation, a semiquantification of the environmental samples was achieved in the calibration ranging from 1 to 10,000 ng/L. A LOD of 1.5 ng/L and a LOQ of 5.0 ng/L were determined.

## 3 Results and Discussion

### 3.1 Identification of the Estrogenic Main Driver

#### 3.1.1 Analysis of the Extracted Leachate

As the planar test systems are optimized for samples dissolved in organic solvents, when silica plates were used, the leachate of the elastomer membrane was enriched by SPE with OASIS HLB 6 cm^3^ (200 mg) using methanol as the elution solvent. The extracts showed two estrogenic active signals in the p-YES after chromatographic separation on the HPTLC plate (*R*_f_-values: 0.41 and 0.29, Figure 2). The blank sample of the method showed no estrogenic activity.

**Figure 2:**
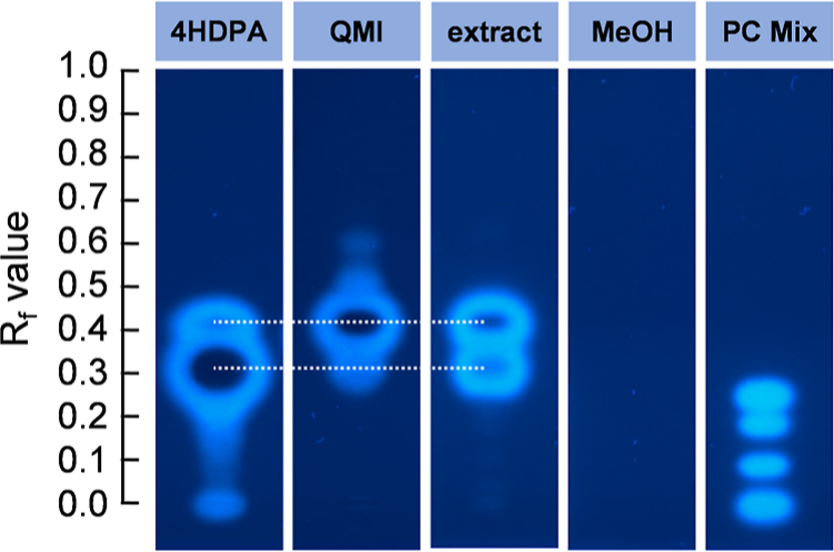
p-YES signals corresponding to active fractions of 4HDPA (2 μg), QMI (2 μg), the extract (10 μL of 5× REF), MeOH solvent blank (5 μL), and positive control using a mixture of four estrogens (PC Mix). A *R*_f_ scale is shown and two dashed lines support the visualization of the equally ran fractions. Retention of estrogens in the PC mix, listed from top to bottom: E1, EE2, E2, and E3 (2 pg; 0.2 pg; 0.2 pg; 20 pg).

It appeared that at least two substances mainly caused the estrogenic effect in the extract. In a recent study, the estrogenic activity of aqueous leached tire particles was also analyzed on a HPTLC plate detecting two estrogenic signals (*R*_f_-values: 0.75 and 0.55, using a different developing method).^22^ The authors stated that several active substances might coelute and therefore lead to a similar retention behavior, causing the estrogenic signals.^22^ Estrogenic effects of leachates of tire particles were already observed via an ERα-CALUX assay, but the responsible substances were not identified.^28^ Whereas in another study, the estrogenicity from leachates of tire particles was examined using an ELRA and YES assay. Here, only the ELRA detected estrogenic effects.^27^

For substance identification in our study, NTS was performed for 1× and 5× REF dilutions of the total SPE extracts of the leachates. In ESI+ and ESI– mode, 2518 and 619 features for the 5×-extract and 1381 and 265 features for the 1×-extract were detected after blank subtraction, respectively. By CSL search,^59^ a total of 14 for ESI+ and 5 for ESI– substances could be tentatively identified (Table S4) as potential candidates. Many of these substances were rubber-related, e.g., 6PPD and *N*-isopropyl-*N*′-phenyl-*p*-phenylenediamine (IPPD), which are used as antioxidants, as well as 2-hydroxybenzothiazole and benzothiazole-2-sulfonic acid, which are TPs of benzothiazoles used as vulcanization accelerators.^10,17^

#### 3.1.2 Identification of Substances in the HPTLC Fractions

To separate the substances, which are possibly responsible for the two signals in the p-YES, the two corresponding fractions (see Figure 2) were extracted from a separately developed HPTLC plate via methanol. The two fractions were reanalyzed with the p-YES, and the results confirmed that both estrogenic substances were successfully extracted (Figures S6 and S7). As expected, the upper fraction caused only one estrogenic signal at the higher *R*_f_ of 0.42. In contrast, both estrogenic signals (*R*_f_ = 0.31 and 0.42) were observed for the lower fraction, which could be explained by a reversible pH-dependent two-electron redox reaction (see discussion in Section 3.1.4).

For the identification of the substances, a further NTS approach was carried out for the two extracted fractions, individually. In ESI+ and ESI–, 991 and 604 features for the upper fraction and 704 and 261 features for the lower fraction were found. Features of the original SPE extract and both HPTLC fractions were compared after processing the data using the R-tool “ntsworkflow”.^65^ A variety of prioritization steps were applied. First, all features, which were detected in the 5× and 1× enriched original extract were filtered, resulting in a total number of 751 and 204 features, for ESI+ and ESI–, respectively. These features were compared to the upper and lower fraction individually, reducing the total number to 62 and 32 features, for ESI+ and ESI–. Second, those features were excluded, which had a higher intensity in the 1× than in the 5× original extract. Furthermore, features detected in the process blank of the HPTLC separation were subtracted, which resulted in a reduced number of 34 and 16 features for ESI+ and ESI–. Only 11 and 5 features of these were present in ESI+ and ESI– for both fractions. These findings could be explained by an incomplete separation and later by a chemical equilibrium between two substances (see discussion below). A further prioritization of features with an intensity >1% compared to the total intensity resulted in a total number of only four features present in both fractions (Table 1). Feature no. 1 was tentatively identified as 4HDPA by the CSL and MS^2^-spectra (Figures 3 and S5). It was finally confirmed with a commercially available authentic reference standard.

**Table 1:** Overview of Candidates for the p-YES Detected in the Extract, Which Were Identified via CSL Search^59^

no.	ESI mode	*m*/*z* [M + H]^+/^ [M – H]^−/^	retention time (min)	intensity in extract	molecular formula	compound name	CAS
1	pos/neg	186.0915/184.0769	9.90	105,450/477	C_12_H_11_NO	4-hydroxydiphenylamine (4HDPA)	122-37-2
2	pos	184.0757	9.92	67,312	C_12_H_9_NO	*N*-phenyl-*p*-benzoquinone monoimine (QMI)	2406-04-4
3	neg	228.0668	7.13	532		unknown	
4	neg	297.1533	14.59	346		unknown	

**Figure 3:**
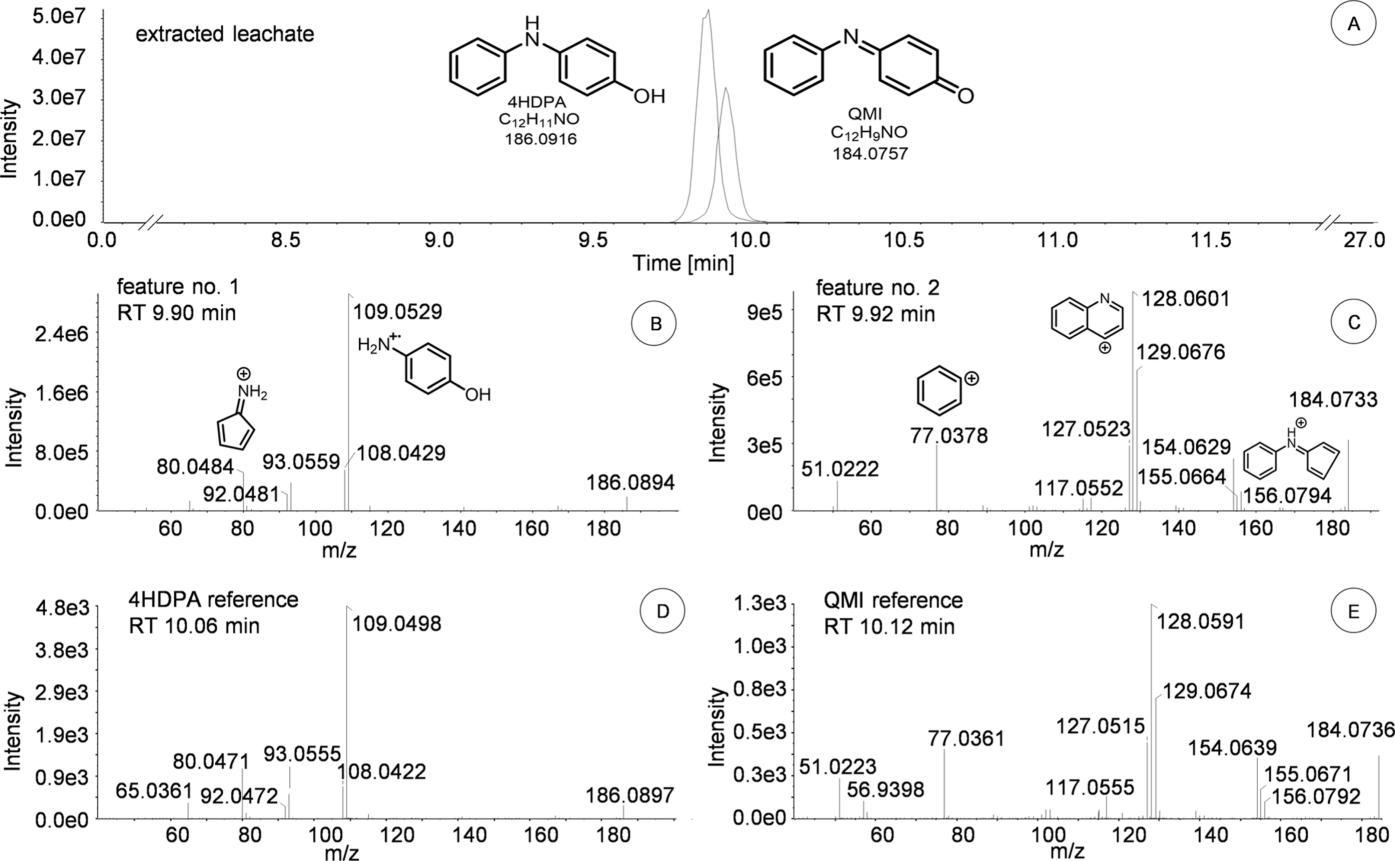
(A) HPLC chromatogram from 9 to 12 min of the extracted leachate showing the XICs *m*/*z* 186.0916 and *m*/*z* 184.0757. The MS^2^-spectra of feature nos. 1 and 2 are shown in: (B) feature no. 1 with *m*/*z* 186.0916, the most prominent MS^2^ fragments, and a retention time (RT) of 9.90 min; (C) feature no. 2 with *m*/*z* 184.0757, MS^2^ fragments, and a RT of 9.92 min; (D) MS^2^-spectra of the reference standard 4HDPA with a RT of 10.06 min; and (E) MS^2^-spectra of the synthesized standard QMI with a RT of 10.12 min.

The substance 4HDPA has been reported to be a major hydrolysis product of 6PPD and IPPD,^14,24,26,69,70^ which are common antioxidants used for rubbers. Both compounds were also detected in the original extracts (Table S4). The *m*/*z* of feature no. 2 was 184.0757 with a RT of 9.92 min. It was tentatively identified as QMI (CL3), which is an oxidation product of 4HDPA.^24,26,69–71^ A compound with the same mass was formerly described by Grasse et al.^24^ as well as by Seiwert et al.^26^ Due to the lack of a commercially available reference standard, we synthesized QMI^47^ and confirmed the chemical structure by NMR (Figure S1). By comparing MS^1^, MS^2^-spectra, and RT with the synthesized standard, feature no. 2 could be unambiguously identified (CL1) as QMI. In the MS^2^-spectra, the most prominent *m*/*z* of 156.0784 and 128.0601 are probably formed through the loss of CO and C_3_H_4_O, respectively, and correspond to the formulas C_11_H_10_N^+^ and C_9_H_6_N^+^. The *m*/*z* of 77.0378 corresponds to the phenyl ring (C_6_H_5_^+^) of QMI (more details supplied in Figure S5). Figure 3 shows the chromatogram from the extracted leachate with the respective MS^2^-spectra of 4HDPA and QMI alongside its reference and synthesized standards.

#### 3.1.3 Verification and Confirmation of Identified Substances

The estrogenic activity and retention behavior on the HPTLC plate were analyzed with the p-YES (Figure 2) to determine whether the identified substances 4HDPA and QMI are responsible or at least contribute to the two active spots of the original extract of the leachate. The solvent blanks did not exhibit any estrogenic effect.

Surprisingly, the original extracts as well as both standards of 4HDPA and QMI exhibited the same prominent estrogenic signals (*R*_f_-values: 0.41 and 0.29–0.30) at different intensities on the HPTLC plate, although pure standards were applied for both substances. The intensity of both signals of the extracted leachate was quite similar, whereas the upper signal for the QMI was more intense than the lower signal and vice versa for 4HDPA. There are two hypothesis for this behavior, either impurities are present and/or transformation reactions occur in the solutions and/or on the HPTLC plate.^22^ According to Bergmann et al.,^22^ it is reasonable that redox reactions might occur on the HPTLC plate during the analysis of rubber-related standards as many of these compounds are inherently reactive with oxidants such as O_2_ or O_3_ to protect the elastomer material from oxidative degradation. Moreover, we found that both compounds (4HDPA and QMI) were always present in different ratios in their individual stock solutions (details in Supporting Information, Figures S8 and S9). After storing the stock solution and the samples for 14 days in methanol and Milli-Q, the ratio between 4HDPA and QMI shifted, while the sum of their peak areas remained constant (Table S5), suggesting that redox reactions occur between both compounds, as already reported for various other quinones and quinone imines.^71,72^

#### 3.1.4 pH-Dependent Formation of QMI

4HDPA and QMI are a redox couple, which undergoes reversible pH-dependent two-electron oxidation and reduction, respectively^71–76^ (eqs 4 and 5, after Ram et al.^71^). Their redox equilibrium in solutions is considerably influenced by different factors such as pH, O_2_-concentrations,^71,73,77^ and the redox potential of the matrix ingredients.

To assess the pH dependency, experiments with 4HDPA (*c* = 50 μg/L) were performed in the range between pH 5 and 9. Due to the adjustments of the LC autosampler, the experiments had to be performed at 4 °C. The obtained areas of 4HDPA and QMI were normalized against the IS bezafibrate-*d*_4_. In an additional experiment, 4HDPA and QMI were spiked directly to the YES medium at 25 °C to elucidate whether the complex medium containing vitamins, salts, amino acids (lysine and histidine, each *c* = 0.1 g/L), glucose (*c* = 127.1 g/L), copper sulfate (*c* = 2.4 mg/L), and antibiotics (ampicillin and streptomycin, each *c* = 0.7 g/L) (details in Table S7) influences the ratio between both compounds.

The results of the spiking experiments with the YES medium at around pH 5.5 exhibited that 96–98% of 4HDPA was present, regardless if 4HDPA or QMI was spiked (Figure 4A,B). Thus, obviously, QMI was immediately reduced to 4HDPA under the conditions of the YES assay at pH 5.5. Varlamov^78^ reported that the reaction of QMI with H_2_O already leads to the formation of 4HDPA. Furthermore, it is even likely that constituents of the YES medium, such as the monosaccharide glucose, accelerate the reduction of QMI. Therefore, QMI and 4HDPA are a redox couple in which, under the YES assay conditions, primarily 4HDPA (97 ± 1%) was present. This assumption is underlined by the quantification of 4HDPA in the YES medium via standard addition and by the very slow oxidation of 4HDPA at pH 5.5, as shown in Figure 4C.

**Figure 4:**
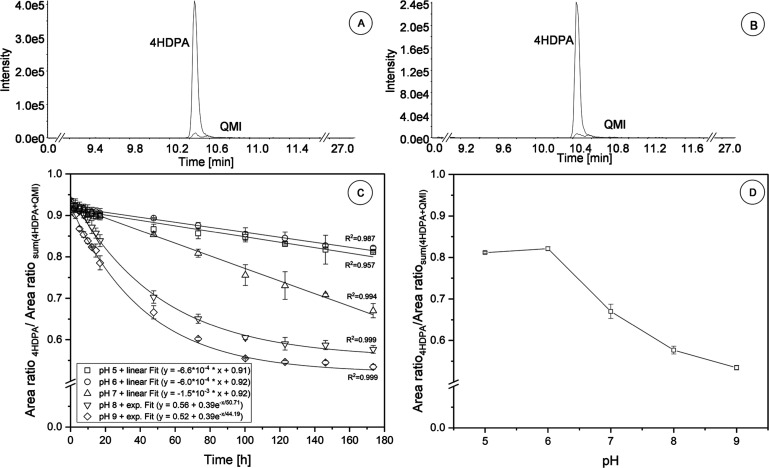
A set of different experiments was performed to explain the detected estrogenicity of QMI. In (A) 4HDPA (*c* = 50 μg/L, *n* = 3) and in (B) QMI (*c* = 50 μg/L, *n* = 3) are spiked into YES conditions. In A and B, the part of the chromatogram from minute 9 to 11 with the XICs *m*/*z* 186.0916 and *m*/*z* 184.0757 is shown. (C) Shows the results of the pH stability experiments of 4HDPA (*c* = 50 μg/L, *n* = 3) spiked in different pH values at 4 °C. Due to limitations in the setup of the pH experiment, the last four time points (100, 123, 146, and 173 h) are only measured in duplicates. The percentage of the area ratios of 4HDPA on the sum of both species normalized against bezafibrate-*d*_4_ is plotted via the time (h). Details about the results of the linear and exponential fits can be found in the Supporting Information, Figures S10 and S14. In (D), the percentage of the area ratio of 4HDPA on the sum of both species at *t* = 173 h is plotted against the pH. Error bars indicate confidence intervals of 95%.

Furthermore, 4HDPA and QMI are the main species of the mass balance in our pH experiments (Figure 4C), since the relative deviation of the sum of both area ratios (4HDPA plus QMI) varied <4% for each replicate and each pH value (Table S6). Thus, QMI is not further transformed, and 4HDPA is only oxidized to QMI.

After 173 h, the percentage of the area ratios of 4HDPA to the total sum of area ratios (4HDPA plus QMI) indicated a linear decrease at pH 5 and 6 from 92 ± 2% and 93 ± 2% to 81 ± 1% and 82 ± 1% with a slope of −6.6 × 10^–4^ (*R* ^2^ = 0.957) and −6.0 × 10^–4^ (*R* ^2^ = 0.987), respectively (Figure 4C), while the ratio of QMI increased accordingly (Figures S10 and S14). At pH 7, the slope of the linear decrease was noticeably higher with −1.5 × 10^–3^ (*R* ^2^ = 0.994) leading to 67 ± 2% of 4HDPA after 173 h. It has to be noted that even after 173 h, an equilibrium could not be reached at pH values ranging from 5 to 7. By further increasing the pH, after 100 h at pH 8, a plateau was reached with 58 ± 1% and at pH 9 with 53 ± 1% 4HDPA. The data of pH 8 and 9 were fitted with an exponential function leading to a *R* ^2^ of 0.999 for both pH values (Figures S13 and S14). Further information about the data of the regression models can be found in Figures S10–S14.

According to Ram et al.,^71^ the pH-dependent formation of the anionic form (4DPA^–^) by deprotonation is probably the limiting step for the oxidation of 4HDPA to QMI^71^ (eq 4). The percentage of 4DPA^–^ increases with an increasing pH (Figure S15). The oxidation of 4DPA^–^ shifts the equilibrium of the deprotonation toward 4DPA^–^.
4

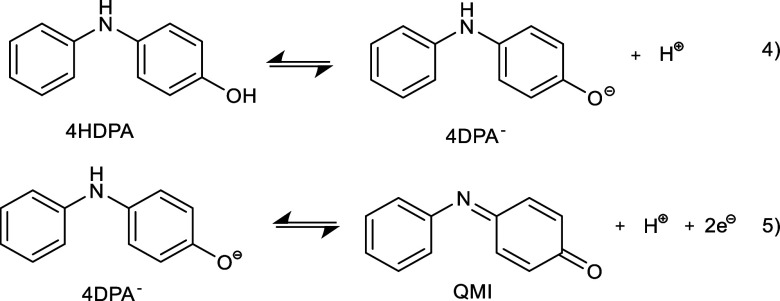




Our results indicate that both 4HDPA and QMI are present in a definite ratio in solutions as well as on the HPTLC plate during the chromatographic run. As a consequence, always two estrogenic signals were observed in the original extract as well as with the reference standards, independent if 4HDPA or QMI was applied on the HPTLC plate. The intensity ratios of 4HDPA and QMI standards were different. It is likely that after the chromatographic run, the incubation of the HPTLC plate containing the YES medium for 3 h at 30 °C leads to a major conversion from QMI to 4HDPA. Thus, it is assumed that the lower signal at *R*_f_ = 0.29 is more intense when a standard solution of 4HDPA is applied and can be attributed to 4HDPA, while the upper signal at *R*_f_ = 0.41 is more intense when QMI is applied and can be attributed to QMI.

The estrogenicity of a molecule primarily depends on the presence of a phenolic OH-group.^79,80^ For example, neither 6PPD nor 6PPD-quinone exhibit estrogenic effects as they lack the requisite OH-group.^22^ The same applies to QMI. Hence, even though the formation and chromatographic separation of both species explains the observed double-signals in the p-YES, the estrogenic effect is most likely caused by 4HDPA due to the presence of the OH-group in *para*-position.^22^ Thus, the estrogenic effect of QMI is very likely overestimated due to the artificial 4HDPA formation under YES conditions. Hence, we assume that 4HDPA is the main driver of the estrogenic effect, while QMI is not estrogenic per se but contributes to the total estrogenic activity after reduction to 4HDPA under YES conditions. In general, it can be assumed that the results of the YES assay must be carefully checked with regard to redox couple compounds, which undergo reversible pH-dependent two-electron redox reactions. However, this needs to be elucidated in detail in future studies.

### 3.2 Contribution of 4HDPA to the Total Estrogenicity of the Leachate

Concentration–response relationships were investigated using the standardized (nonplanar) YES. Since the buffered pH tests already showed that the majority (97 ± 1%) of the applied 4HDPA and QMI is present as 4HDPA (see Section 3.1.4), concentration–response relationships could only be determined for 4HDPA, even when testing a QMI solution. Fitting the effects of the applied concentrations between 160 and 5000 μg/L led to a nondisturbed sigmoidal curve with calculated EC_10_ and EC_50_ values of 330 ± 59 and 820 ± 180 μg/L, respectively (details supplied in Supporting Information, Section 6). For comparison, the EC_50_ of bisphenol A, a well-studied substance leaching from artificial materials, was found to be 302 ± 24 μg/L.^19^ This value is 2.7 times smaller but lies in the same order of magnitude as the results of 4HDPA. In order to determine to which extent the presence of the redox couple of 4HDPA and QMI was responsible for the observed estrogenic effects of the leachate in the YES, 4HDPA was quantified in the leachate via the standard addition approach (Figure 5).

**Figure 5:**
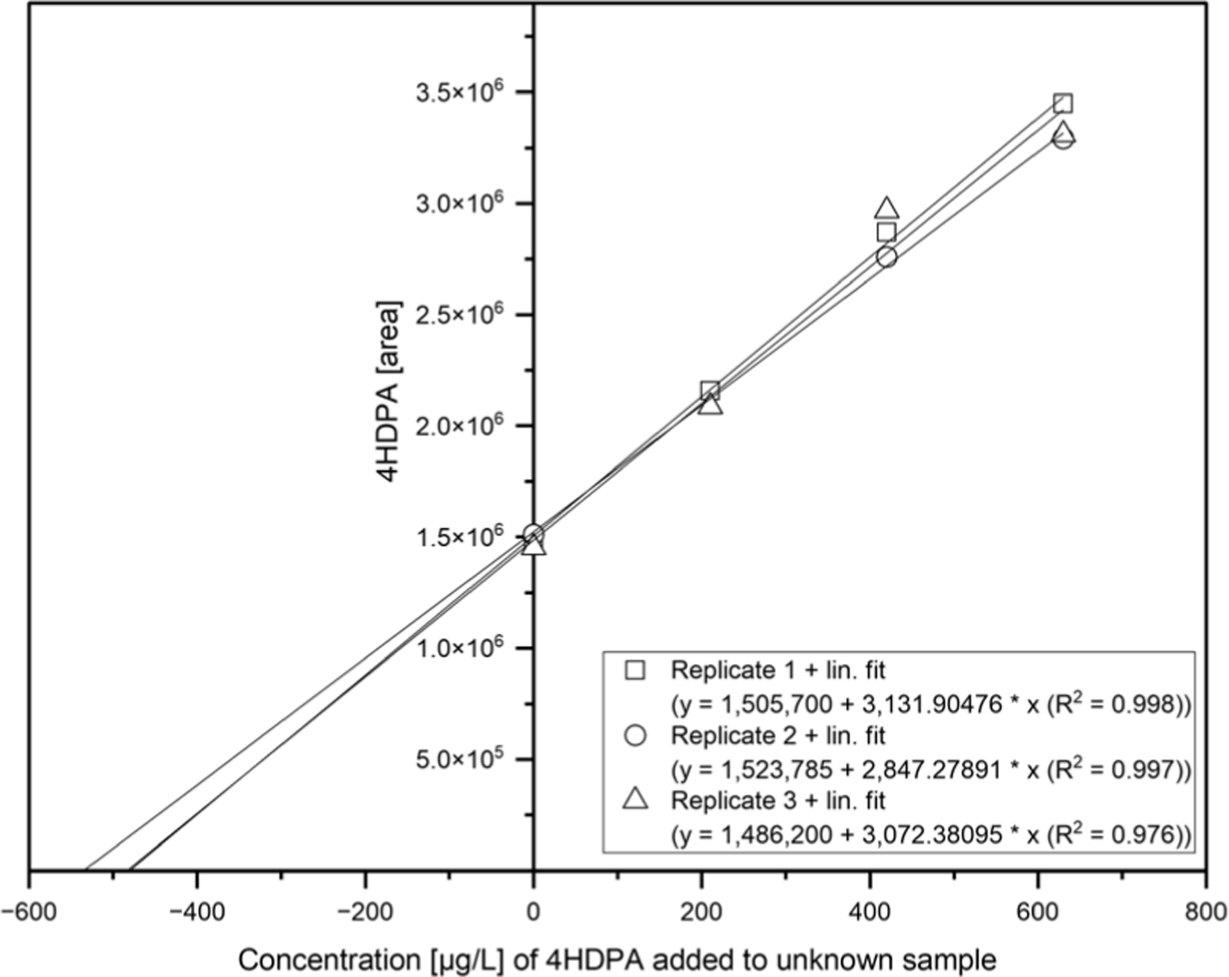
Results of standard addition method of the leachate (*n* = 3) with spiked concentrations of 4HDPA of level 1 = 210, level 2 = 420, level 3 = 630, and level 4 = 840 μg/L, and an injection volume of 1 μL are shown. YES conditions were applied as followed: 20 μL of the leachate, 60 μL of Milli-Q, and 40 μL of YES exposure medium, which corresponds to a dilution factor of 6. Concentrations of the triplicates were calculated using the intersection of the *x*-axis, resulting in a concentration of 3000 ± 170 μg/L.

Afterward, the concentration–response relationship of a dilution series of the leachate was compared with those of the 4HDPA standard. Since the equilibrium is mainly on the side of 4HDPA in the YES assay, causing the estrogenic effect, only 4HDPA was quantified by the standard addition method with the same ratio of leachate and YES medium as used in the YES assay. Thereby, a 4HDPA concentration of 3000 ± 170 μg/L was determined in the leachate. The determined estrogenicity and 4HDPA concentrations were used to calculate the percentage of 4HDPA on the whole estrogenic potential of the leachate.^44,45^ The overall estrogenic potential of the leachate was 140 ± 7 ng/L E2-EQ. By using a relative potency of 4HDPA with 5.7 × 10^–5^ (EC_10_(E2) = 19 ± 3 ng/L and EC_10_(4HDPA) = 329,000 ± 59,000 ng/L) and the concentration of 4HDPA (3000 ± 170 μg/L), 120 ± 30% of the observed estrogenicity is explained by 4HDPA, indicating that this compound is the main driver of the effect in the aqueous leachates of the elastomer investigated. The concentration–response curves corresponding to 4HDPA and the leachate are shown in Figures S16 and S17.

However, in the aquatic environment as well as in biota cells, different ratios of 4HDPA and QMI can be present, depending on the pH and the redox conditions. This might influence the estrogenic effects. As it is known that an unhindered OH-group in *para*-position is usually a prerequisite for the interaction with the estrogen receptor,^24^ it can be assumed that the activity of QMI is considerably lower compared to 4HDPA. Hence, the EC-values determined by the YES reflect a worst-case scenario where under the experimental conditions, mainly 4HPDA is present. However, hydroquinones, as well as hydroquinone imines, can be oxidized to the corresponding quinones by monooxygenases or peroxidases, metal ions, and also by molecular oxygen in cells.^77,81,82^ Since it would be nearly impossible to assess the ratio of the species present in the cells, the comparison of the sum of the environmental concentrations of 4HDPA and QMI with the EC-values determined for 4HDPA is essential. However, further research is needed to elucidate the equilibrium of the two substances in the aquatic environment, as well as under physiological conditions, for an improved assessment of the ecotoxicological potential of the 4HDPA/QMI redox couple.

### 3.3 Environmental Occurrence of 4HDPA and QMI

In this study, 4HDPA was retrospectively quantified in water samples using the online NTS portal, which contains data provided by 8 German federal states.^64,65^ However, it has to be noted that this is only a first estimation of the environmental concentrations since a reliable target method was not available. Therefore, after the development of a robust target method for 4HDPA and QMI, an extended monitoring campaign for German rivers and streams is foreseen. Using the NTS portal in four monitoring stations, 4HDPA was found in the years 2019 and 2021 in German rivers (details in Table S11). These stations are located at the Rhine (Koblenz), Parthe (Leipzig), Ölsabach (Thrandt), and Vereinigte Weisseritz (Cotta). The estimated concentrations ranged between 7 and 14 ng/L (Figures S18 and S19). QMI was not found, neither by the exact mass nor by the MS^2^-spectra in the NTS-portal database. QMI was only tentatively detected in lab-scale experiments (CL3)^24,26^ and CL4.^8^ As we have no available information about detection limits and the ionization sensitivity, no conclusions can be drawn about the presence of QMI. We retrospectively evaluated NTS-data from samples of the Nordumfluter, where a new rubber dam was built. 4HDPA semiquantified concentrations of 20 ± 2 ng/L were found in grab samples upstream and downstream (approximately 1.5 km) of the dam. They are in a range similar to values reported in the NTS-portal. However, in the water inside the rubber dam, both species could be detected and retrospectively semiquantified as 72 ± 5 μg/L. These findings underline that leaching of 4HDPA from elastomer membranes and tires is a source for environmental contaminations. Seiwert et al.^26^ reported concentrations of 4HDPA ranging between 23 ± 25 ng/L and 70 ± 10 ng/L in the influent of a wastewater treatment plant (WWTP) at dry weather and after snowmelt, respectively. In addition, they found 18 ± 14 ng/L in the WWTP effluent for snowmelt and rainfall. Zhao et al.^7^ reported concentrations of 4HDPA in roadway runoff after storm events with slightly higher concentration levels (150 ± 100 ng/L and 110 ± 50 ng/L). However, the authors also pointed out a limited environmental stability of 4HDPA in surface waters, e.g., due to phototransformation (half-life of 1.3 h in lab-experiments in water).^7^ They attributed the origin of 4HDPA to the transformation of the antioxidant 6PPD, which is used in all kind of tires,^4,7^ elastomer membranes, and many other rubber materials.

### 3.4 Environmental Impact of 4HDPA and QMI

In this study, an EDA approach was successfully applied to identify the main drivers of estrogenicity from aqueous leachates of an elastomer membrane applied in, e.g., water-filled rubber dams. For the first time, the redox couple of 4HDPA and QMI was identified as an estrogenic driver. Even though 4HDPA and QMI can be assumed to be ubiquitously released from various elastomers (e.g., tires and tire particles) by the transformation of PPDs, knowledge about their occurrence in the aquatic environment and (eco)­toxicity studies is scarce. According to the ECOTOX Knowledgebase and ChEMBL^83^ (accessed 20th January 2025), 4HDPA was shown to influence the swimming behavior of *danio rerio*.^84^ For 4HDPA, the CompTox Dashboard^85^ (accessed 20th January 2025) provided data regarding bioactivity in several molecular mechanisms and acute toxicity for mice (LD_50_ 1600 mg/kg oral) and rats (LD_50_ 3300 mg/kg, oral). No information is available for QMI in all three databases.

So far, the literature data and the semiquantitative determination in this study showed concentrations of 4HDPA in WWTP effluents and surface waters more than 1000 times lower than our estimated EC_10_ of 4HDPA (330 ± 59 μg/L). Nevertheless, considering the existing environmental xenoestrogenic pollution,^86,87^ 4HDPA might contribute to the total estrogenic, i.e., ER-agonistic potential in the environment. Moreover endocrine active substances can trigger chronic effects in concentrations lower than their acute effect values,^31^ and thus chronic effects of 4HDPA as well as more comprehensive data on the occurrence, sources, and fate need to be determined in follow-up studies. Moreover, the complex equilibrium of 4HDPA with QMI needs to be further elucidated since currently we cannot exclude the possibility that QMI might pose potential risks. Although specific information on the toxicity of QMI is lacking,^24^ it most likely causes cytotoxicity, immunotoxicity, and carcinogenesis, as well as damage proteins and other cellular macromolecules in elevated concentrations. This might be caused through the formation of reactive oxygen species^74,77^ such as superoxides, hydrogen peroxide, and hydroxyl radicals^74^ similar to other quinones and quinone imines^76,77,82^ due to their high electrophilicity and redox potential.^46,76,82^ Moreover, they are “soft” electrophiles^74–77,82^ and react as Michael acceptors, therefore, they might even alkylate DNA. Cytotoxic effects beside estrogenicity were also observed in the p-YES experiments with 4HDPA, QMI, and the extracts, as indicated by the ring patterns shown in Figure 2. The lacking estrogenic signals in the center are attributed to dead cells and therefore indicate cytotoxicity.^49,88^ For 4HDPA, this could be due to autoxidation to the corresponding quinone imine,^76^ such as QMI.

In conclusion, the results of this study highlight that the analysis and interpretation of the occurrence and toxicity of hydroquinones and hydroquinone imines must be considered with special care. Therefore, the results can be considerably influenced by the specific ratio of the redox couples in the aquatic environment as well as under special test conditions. Moreover, considering the high number of compounds eluting from rubber materials, further EDA studies are crucial to identify the drivers of other end points observed from leachates, such as antiandrogenic or dioxin-like effects.

## Supplementary Material



## Notes

Detailed information on substances, YES exposure medium, QMI synthesis, material composition, and chemical analysis; results of bioassays, bioanalytical equivalent concentrations, pH experiments, and standard addition; chromatograms of spiking experiments and stock solutions and MS^2^-spectra; and identified substances using the in-house CSL and NTS-portal search (PDF)
