# Supplementary material for: Deciphering the Estrogenic Activity of Aqueous Leachates from Elastomers by Effect-Directed Analysis
Source: Environ Sci Technol. 2025 Sep 30;59(40):21442–53. doi: 10.1021/acs.est.5c05987 (PMC12529948; doi:10.1021/acs.est.5c05987)
Supplement: Supplementary file 1 [file es5c05987_si_001.pdf]

Deciphering the Estrogenic Activity of Aqueous Leachates from Elastomers by Effect-Directed Analysis

Supplementary Information

Rebecca Süßmuth,† Timothy Rosenberger,† Peter Schweyen, Georg Dierkes, Anna Maria Bell, Arne Wick, Sebastian Buchinger, and Thomas A. Ternes*

Federal Institute of Hydrology, Am Mainzer Tor 1, 56068 Koblenz, Germany

†These authors share first authorship and contributed equally to this work.

*corresponding author: ternes@bafg.de

Summary: 23 pages / 8 sections / 19 figures / 11 tables

Table of Content

1. Information on chemicals and samples S5

1.1 Chemicals and Reagents S5

1.2 Synthesis of N-phenyl-p-benzoquinone monoimine (QMI) S6

1.3 Details on material elastomer membrane S7

2. Chemical analysis S8

2.1 Details on LC gradient used for non-target screening approach S8

2.2 Standard addition: statistical data of the linear regression S9

3. Identification of candidates using the NTS approach S10

3.1 Identified candidates in the total aqueous leachate S10

3.2 MS^2-Spectra of 4HDPA (feature no. 1) and QMI (feature no. 2) S12

4. Results of bioassays S12

4.1 Extracted HPTLC Fractions S12

5. Equilibrium between QMI and 4HDPA S13

5.1 Experiments using different solvents S13

5.2 pH experiments S14

5.3 Ingredients of YES exposure medium S17

6. Quantification of estrogenic potential S18

6.1 Effect data of the Yeast Estrogen Screens S18

7. NTS-portal search S20

8. References S22

List of figures

Figure S1: ^{13}C-NMR for QMI S6

Figure S2: ^{13}C-NMR for 4HDPA S7

Figure S3: Picture of the used elastomer membrane S7

Figure S4: FT-IR spectra of the white/inner fibers and the red/outer fibers S8

Figure S5: The MS^2 spectra of feature no. 1 and 2 .... S12

Figure S6: HPTLC Image of the fractionized extract S12

Figure S7: p-YES image of the re-analyzed extracted extract fractions. S13

Figure S8: Chromatogram of 4HDPA spiked in MeOH and ultrapure water S13

Figure S9:. Chromatogram of QMI spiked in MeOH and ultrapure water S14

Figure S10: pH stability experiments of 4HDPA spiked in pH 5. .... S14

Figure S11: pH stability experiments of 4HDPA spiked in pH 6. .... S15

Figure S12: pH stability experiments of 4HDPA spiked in pH 7. .... S15

Figure S13: pH stability experiments of 4HDPA spiked in pH 8. .... S16

Figure S14: pH stability experiments of 4HDPA spiked in pH 9. .... S16

Figure S15: Distribution of the deprotonated anion $4DPA^-$ depending on pH and the pK_a value of 4HDPA. S17

Figure S16: Concentration-response curves and raw data. S18

Figure S17: Concentration-response curves and raw data S20

Figure S18: Average MS^2 spectrum of 4HDPA in the NTS-portal[11,12]. S20

Figure S19: Semi-quantified concentrations of 4HDPA S22

List of tables

Table S1: Information on all chemicals and solvents. S5

Table S2: Details on LC-MS analysis S8

Table S3: Fitting data of the standard addition approach. S9

Table S4: Identified substances in the NTS approach S10

Table S5: Area ratios and percentage of QMI on sum of 4HDPA and QMI S14

Table S6: Means and confidence interval (CI) at 95% of sum of areas of 4HDPA and QMI. S14

Table S7: Ingredients of the exposure medium. S17

Table S8: Underlying Data for the calculation of concentration-response relationships of 4HDPA in the YES. S18

Table S9: Underlying Data for the calculation of concentration -response relationships of QMI in the YES S19

Table S10: Estimated effect concentrations for 4HDPA and QMI S19

Table S11: Data table exported from the NTS-portal S21

1. Information on chemicals and samples

1.1 Chemicals and Reagents

Table S1: Information on all chemicals and solvents used in the study.

Substance/ solvent	CAS	Supplier
Estrone (E1, ≥ 99%)	53–16-7	Sigma-Aldrich
17β-Estradiol (E2, ≥ 98%)	50–28-2	Sigma-Aldrich
17α-Ethinylestradiol (EE2, ≥ 98%)	57–63-6	Sigma-Aldrich
Estriol (E3, ≥ 97%)	50–27-1	Sigma-Aldrich
4-Hydroxydiphenylamine (>95%)	122-37-2	TCI Germany
4-Methylumbelliferyl-β-D-galactopyranoside (MUG)	6160–78-7	Sigma-Aldrich
Bezafibrate-d4	1189452-53-6	TRC
Iopromide-d3	1189947–73-6	TRC
Olmesartan acid-d6	1185144–74-4	Biozol
Silver carbonate on celite	534-16-7	Sigma-Aldrich
di-Sodium tetraborate anhydrous (>98%)	1330-43-4	Merck
di-Sodium hydrogen phosphate dihydrate (>99%)	10028-24-7	Carl Roth
Sodium dihydrogen phosphate dihydrate (>99%)	13472-35-0	Carl Roth
Bis-(2-hydroxyethyl)-amino-tris-(hydroxymethyl)-methane (>99%)	6976-37-0	Carl Roth
Sodium acetate (>99%)	127-09-3	Carl Roth
Hydrochloric acid 25%	7647-01-0	Supelco
Sodium hydroxide	1310-73-2	Sigma Aldrich
Acetone (Picograde)	67–64-1	Promochem
Acetonitrile (hypergrade for LC-MS)	75-05-8	Supelco
Ethanol (≥ 99.8%,)	64–17-5	Riedel-de-Haen
Ethyl acetate (99,5%)	141-78-6	Promochem
Formic acid (Suprapur)	64-18-6	Merck
n-Heptane (Picograde)	142–82-5	Promochem
Methanol (≥ 99.9%)	67–56-1	Merck
Toluene (≥99.9%)	108-88-3	Merck

1.2 Synthesis of N-phenyl-p-benzoquinone monoimine (QMI)

Synthesis was performed according to Baragona et al.[1] using Fétizons reagent[2]. First, 2.00 mmol of 4-hydroxydiphenylamine (4HDPA) was weighed in a round-bottom flask (m = 370 mg) and 80 mL of toluene and 2.11 mmol of silver carbonate on celite (m = 1.2 g) were added in one portion. The mixture was stirred for 45 min at 0 °C. The reaction was carried out under a nitrogen steam and with oven-dried glass ware. The reaction mixture was decanted and evaporated under reduced pressure. QMI was obtained as an orange solid and confirmed using Spinsolve 80^{ULTRA} nuclear magnetic resonance (NMR) (Magritek, Aachen, Germany) in deuterated chloroform ($CdCl_3$). The ^{13}C-NMR for QMI is shown in Figure S1 and in comparison, for 4HDPA in Figure S2.

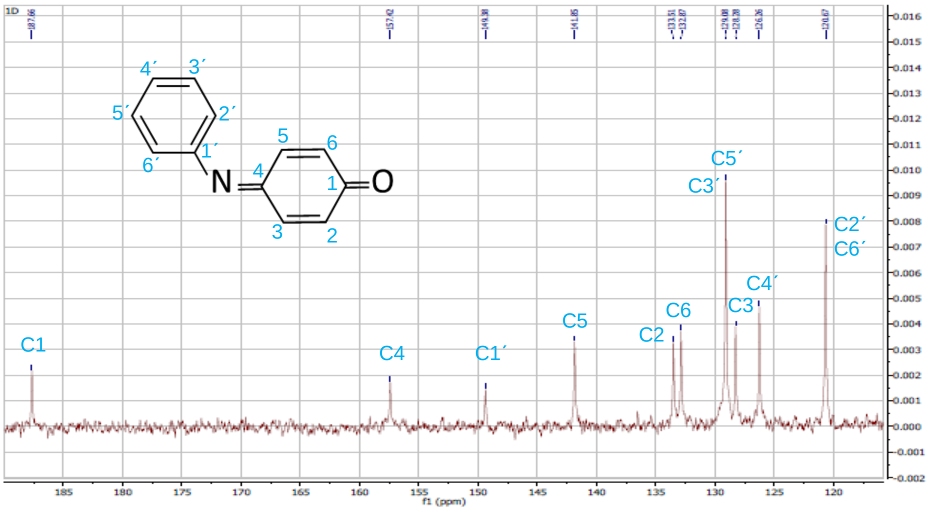

Peaks:
120: C2´C6`
126: C4´
128: C3
129: C3´C5`
132: C6
133: C2
141: C5
149: C1´
157: C4
187: C1

Figure S1: ^{13}C-NMR for QMI in $CdCl_3$ with δ = 187 (C1), 157 (C4), 149 (C1'), 141 (C5), 133 (C2), 132 (C6), 129 (C3', C5'), 128 (C3), 126 (C4'), 120 (C2', C6') according to Adesina[3] showing amplitude against chemical shift.

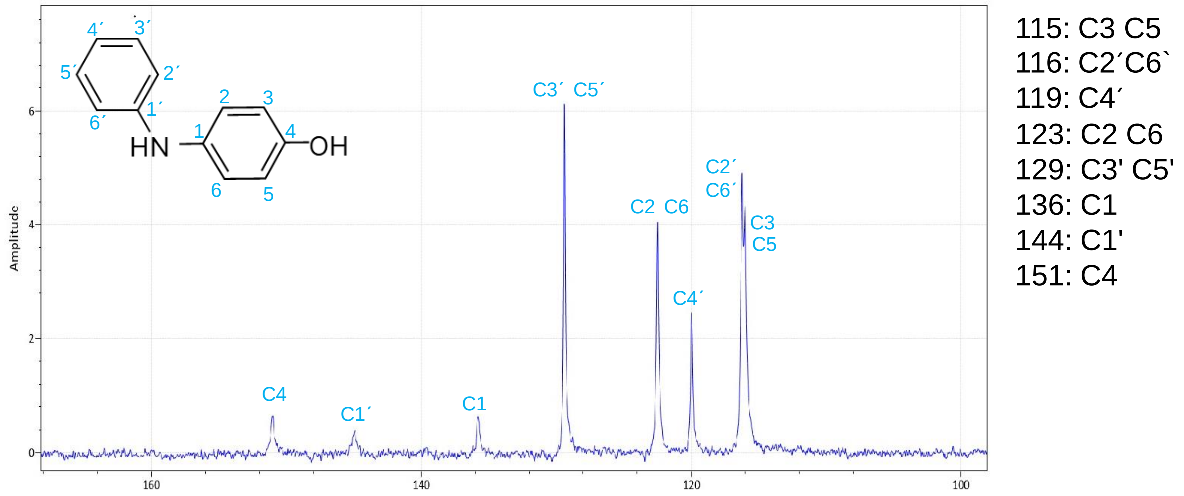

Figure S2: ^{13}C-NMR for 4HDPA in $CdCl_3$ with δ = 151 (C4), 144 (C1'), 136 (C1), 129 (C3', C5'), 123 (C2, C6), 119 (C4'), 116 (C2', C6'), 115 (C3, C5) according to Adesina[3] showing amplitude against chemical shift.

1.3 Details on material elastomer membrane

The elastomer membrane consists of three layers of an elastomer material and three layers of fibers/fabric (Figure S3). Layer 2 was defined as the layer between the fiber layers as it was very difficult to separate the elastomer from the fabric.

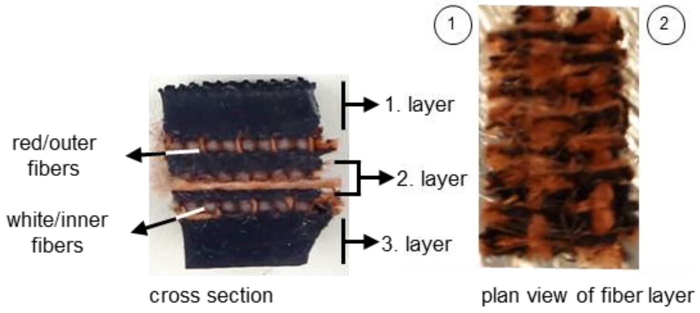

Figure S3: Picture of the used elastomer membrane, 1) cross section and 2) plan view onto the fibers.

A piece of the membrane was dissected to characterize the different layers. The three elastomer layers were analyzed via pyrolysis-gas chromatography-mass spectrometry (Py-GC/MS) using a Multi-Shot Pyrolyzer EGA/PY-3030D (Frontier Laboratories, Saikon, Japan) and an Auto-Shot sampler AS-1020E (Frontier Laboratories, Saikon, Japan) which were connected to a 7890B GC System (Agilent, Santa Clara, CA, USA). Separation was achieved on an Ultra ALLOY® UA-5(MS/HT) metal capillary column (Frontier Laboratories, Saikon, Japan). For detection, a 5977B MSD mass spectrometer (Agilent, Santa Clara, CA, USA) was used. Details on the parameters of

the analysis can be found in Dierkes et al.[4]. The fibers were divided in red/outer and white/inner fibers and analyzed via Fourier-transform infrared spectroscopy (FTIR) using the Frontier FT-IR (Perkin Elmer, Massachusetts, United States).

The results for the elastomer layers show that all three layers consist of a blend of ethylene propylene diene (EPDM) with a styrene component and for the fibre layer that the inner and outer fibers consist of polyethylene terephthalate (PET) and nylon (PA), respectively (Figure S4).

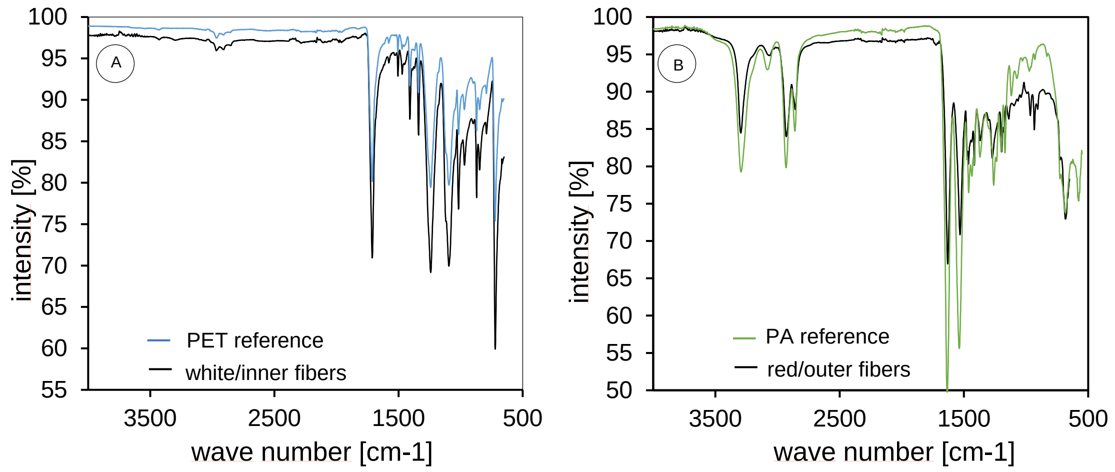

Figure S4: FT-IR spectra of A) the white/inner fibers (black line) and B) the red/outer fibers (black line) including references of PET (blue line) and PA (green line) showing the intensity [%] against the wave number [cm-1].

2. Chemical analysis

2.1 Details on LC gradient used for non-target screening approach

Table S2: Details on LC-MS analysis, gradient using Milli-Q with 0.1 % formic acid (Eluent A) and acetonitrile with 0.1% formic acid (Eluent B).

**Time** [min]	**Flow rate** [µL/min]	**Eluent A** [%]	**Eluent B** [%]
0	300	98	2
1	300	98	2
2	300	80	20
16.5	300	0	100
22	300	0	100
22.1	300	98	2
27.0	300	98	2

2.2 Standard addition: statistical data of the linear regression

Table S3: Fitting data of the standard addition approach according to DIN 32633[5] obtained from Origin 2024 using the formula y = a + b*x.

	triplicate 1	triplicate 2	triplicate 3
Intersection with the y-axis	1,505,700 ± 35,151.17352	1,523,785.71429 ± 46,099.54845	1,486,200 ± 134,796.81005
Intersection with the x-axis	-480.76175 ± 23.70207	-535.17262 ± 34.18924	-483.72908 ± 92.9709
Slope	3,131.90476 ± 89.47187	2,847.27891 ± 105.45479	3,072.38095 ± 343.10441
Sum of the error squares	$3.53*10^{9}$	$2.29*10^{9}$	$5.19*10^{10}$
Pearson R	0.999	0.999	0.988
R-squared (COD)	0.998	0.999	0.976

3. Identification of candidates using the NTS approach

3.1 Identified candidates in the total aqueous leachate

Table S4: Identified substances in the extract (1x and 5x) using the NTS approach and comparison by in-house collective spectral library (CSL)[6] with the according m/z and retention times (RT) of the features and in comparison the data from the CSL.

		extract (1x)		extract (5x)		annotation CSL					
**Mean m/z**	**Mean RT** [min]	**m/z**	**RT** [min]	**m/z**	**RT** [min]	**m/z data**	**RT data** [min]	**Name**	**CAS**	**Score**	**Mode**
114.0902	5.15	114.0902	5.15	0	0	114.0902	5.15	Caprolactam	105-60-2	996	pos
136.0209	8.48	0	0	136.0209	8.48	136.0209	8.48	Benzothiazole	95-16-9	996	pos
150.0023	7.76	150.002	7.77	150.0027	7.75	150.0027	7.75	2-Hydroxybenzothiazole	934-34-9	1000	neg
152.0155	7.77	152.0156	7.78	152.0154	7.76	152.0154	7.76	2-Hydroxybenzothiazole	934-34-9	893	pos
186.0905	9.9	186.0902	9.9	186.0908	9.91	186.0908	9.91	4-Hydroxydiphenylamine	122-37-2	1000	pos
192.1731	5.98	192.1732	5.98	192.1731	5.97	192.1731	5.97	Benzyl-triethylammonium	16652-03-2	999	pos
207.0126	6.14	0	0	207.0126	6.14	207.0126	6.14	2-Naphthalenesulfonic acid	120-18-3	913	neg
212.1168	5.75	212.1168	5.75	0	0	212.1168	5.75	1,3-Diphenylguanidine	102-06-7	957	pos
213.9637	5.69	213.9632	5.71	213.9641	5.68	213.9641	5.68	Benzothiazole-2-sulfonic acid	941-57-1	1000	neg
221.0737	9.14	221.0741	9.14	221.0733	9.13	221.0733	9.13	2-Morpholin-4-yl-benzothiazole	4225-26-7	969	pos
225.1007	5.57	0	0	225.1007	5.57	225.1007	5.57	2-(2-Methoxyphenyl)-1H-Benzimidazole	6528-85-4	987	pos
225.1957	10.65	225.1957	10.65	225.1957	10.65	225.1957	10.65	1,3-Dicyclohexylurea	2387-23-7	962	pos
227.1535	7.18	227.1537	7.18	227.1533	7.18	227.1533	7.18	N-Isopropyl-N'-phenyl-p-phenylenediamine	101-72-4	1000	pos
261.1373	14.08	261.1371	14.08	261.1374	14.08	261.1374	14.08	N,N`-diphenyl-p-phenylenediamine	74-31-7	1000	pos

265.1488	14.19	265.1484	14.18	265.1492	14.17	265.1492	14.17	Dodecyl sulfate	151-41-7	1000	neg
269.1993	9.11	0	0	269.1993	9.11	269.1993	9.11	N-(1,3-Dimethylbutyl)-N`-phenyl-p-phenylenediamine	793-24-8	1000	pos
293.1793	16.83	293.1787	16.82	293.1795	16.81	293.1795	16.81	Myristyl sulfate	4754-44-3	1000	neg
407.3344	16.59	407.3345	16.6	407.3344	16.58	407.3344	16.58	Laureth-5	3055-95-6	751	pos
577.1331	14.4	577.1325	14.39	577.1337	14.4	577.1337	14.4	Ethylene terephthalate cyclic trimer	7441-32-9	919	pos

3.2 MS^2-Spectra of 4HDPA (feature no. 1) and QMI (feature no. 2)

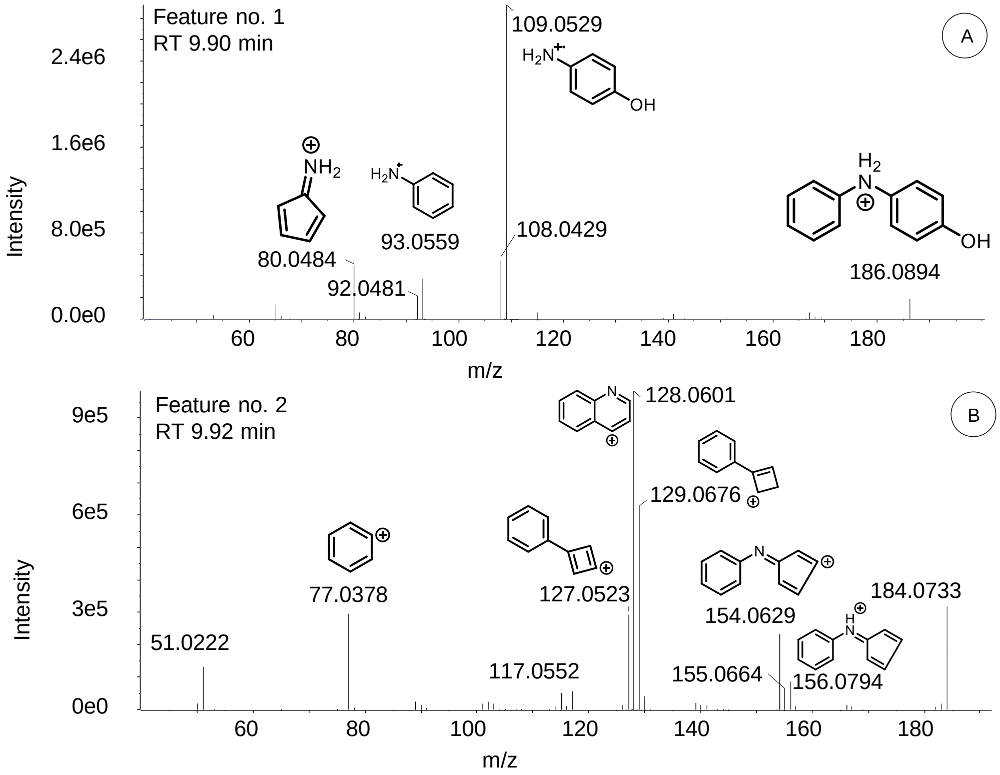

Figure S5: The MS^2 spectra of feature no. 1 and 2 are shown in: A) feature no. 1 with *m/z* 186.0916, prominent MS^2 fragments and a retention time (RT) of 9.90 min and B) feature no. 2 with *m/z* 184.0757, MS^2 fragments and a RT of 9.92 min.

4. Results of bioassays

4.1 Extracted HPTLC Fractions

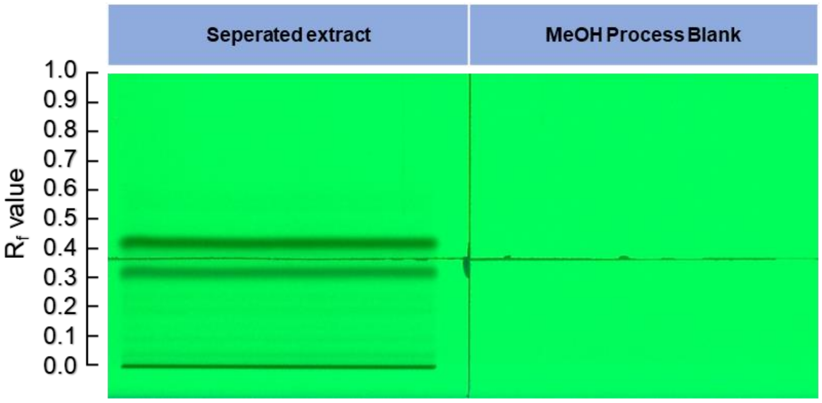

Figure S6: HPTLC Image of the fractionized extract. Horizontal cut at 30.5 mm, vertical cut at 100 mm.

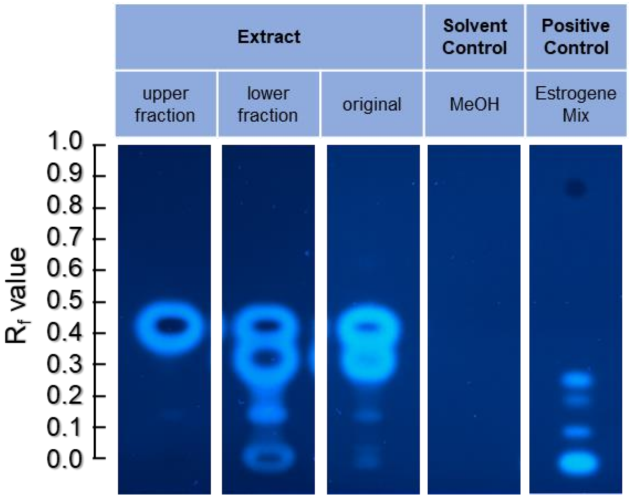

Figure S7: p-YES image of the re-analyzed extracted extract fractions. From left to right, separated extract upper fraction, lower fraction, original extract (10 µL of 5x REF), MeOH solvent blank and positive control.

5. Equilibrium between QMI and 4HDPA

5.1 Experiments using different solvents

<u>4HDPA</u>

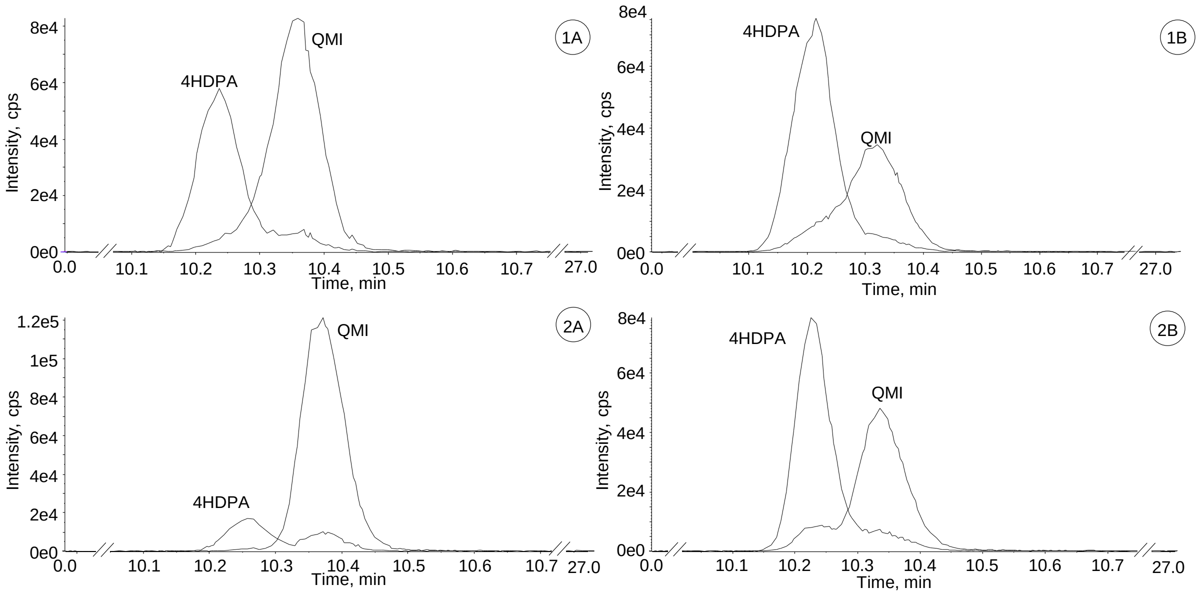

Figure S8: 4HDPA (c = 5 µg/L, injection volume = 10 µL) spiked in MeOH (1) and ultrapure water (Milli-Q) (2), analyzed directly after spiking (A) and after storing for 14 d at -20°C in the dark (B). Results are showing two peaks, for 4HDPA (right) and QMI (left).

Table S5: Area ratios and percentage of QMI on sum of 4HDPA and QMI

Solvent	Time	4HDPA	QMI	Sum	QMI/Sum	after 14 d
		Area ratio	Area ratio	Area ratio	[%]	[%]
MeOH	0 d	4.4	6.5	10.9	60	
	14 d	6.1	3.6	9.7	37	89
Milli-Q	0 d	2.0	9.3	11.3	82	
	14 d	6.0	4.9	10.9	45	96

QMI

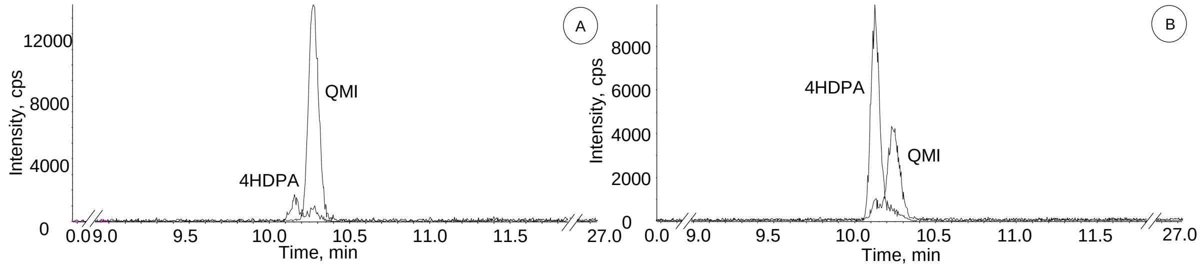

Figure S9: QMI (c = 1 µg/L, injection volume = 10 µL) spiked in Milli-Q, analyzed directly after spiking (A) and after storing for 14 d at -20°C in the dark (B). Results are showing two peaks, for 4HDPA (right) and QMI (left).

5.2 pH experiments

Table S6: Means and confidence interval (CI) at 95% of sum of areas of 4HDPA and QMI for each replicate of each pH value.

Replicate	pH 5		pH 6		pH 7		pH 8		pH 9	
	mean	CI (95%)	mean	CI (95%)	mean	CI (95%)	mean	CI (95%)	mean	CI (95%)
1	188	6	199	4	182	5	170	5	171	5
2	187	7	198	7	186	7	180	5	175	5
3	194	7	195	5	204	6	203	7	189	6

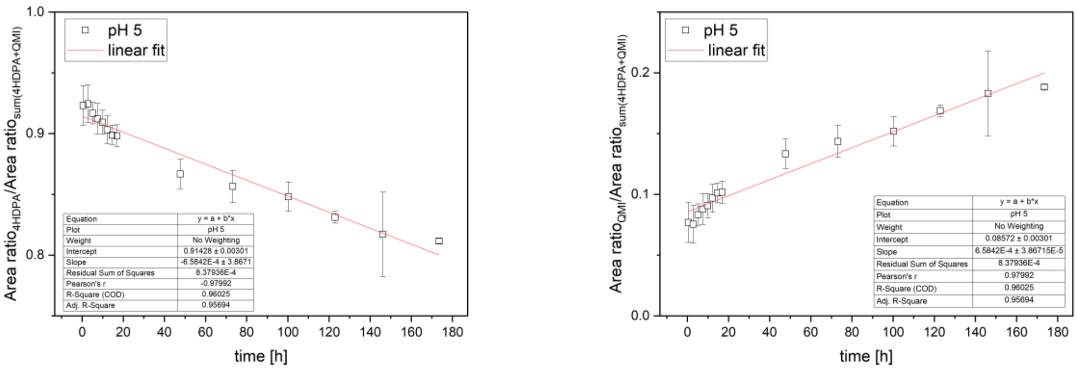

Figure S10: The results of the pH stability experiments of 4HDPA (c = 50 µg/L) spiked in pH 5 at 4°C in triplicates are shown. Due to limitations in the set-up of the pH experiment, the last four time points (100, 123, 146 and 173 h) are only measured in duplicates. The percentage of the area ratios of 4HDPA (left side) and of QMI (right side) on the sum of both species is plotted via the time (h). Error bars indicate confidence intervals of 95%. The red line indicates the linear regression in the form of y = a + b*x. Values are given in the figure.

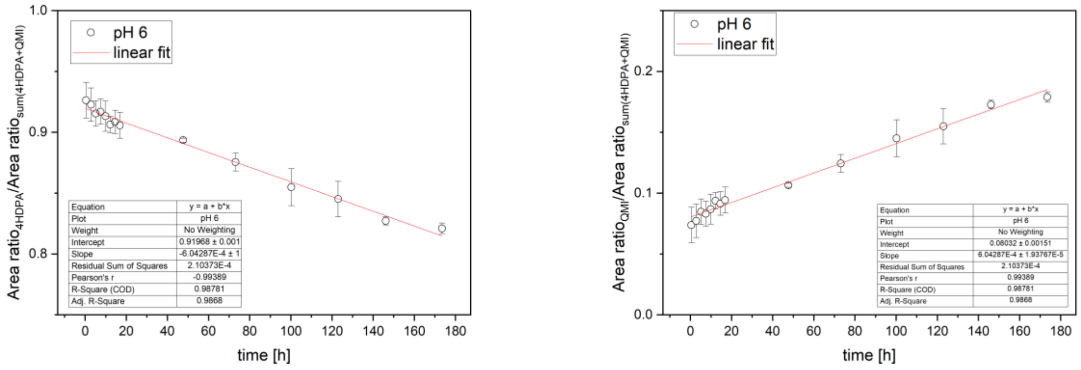

Figure S11: The results of the pH stability experiments of 4HDPA (c = 50 µg/L) spiked in pH 6 at 4°C in triplicates are shown. Due to limitations in the set-up of the pH experiment, the last four time points (100, 123, 146 and 173 h) are only measured in duplicates. The percentage of the area ratios of 4HDPA (left side) and of QMI (right side) on the sum of both species is plotted via the time (h). Error bars indicate confidence intervals of 95%. The red line indicates the linear regression in the form of y = a + b*x. Values are given in the figure.

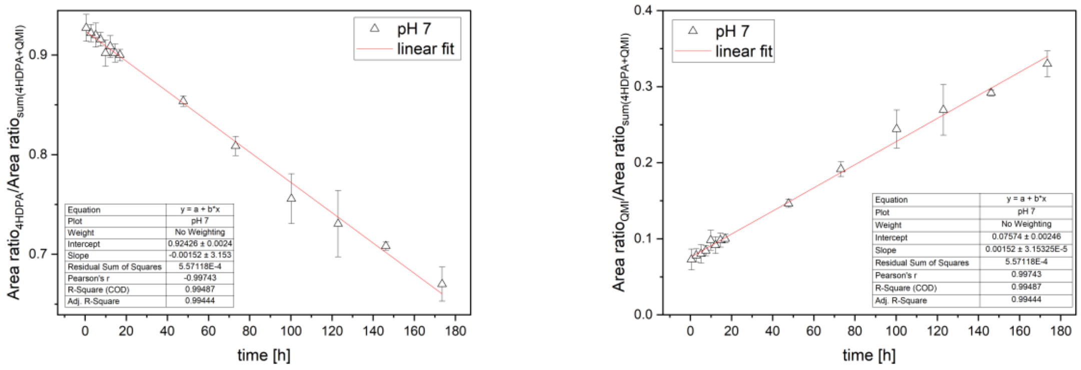

Figure S12: The results of the pH stability experiments of 4HDPA (c = 50 µg/L) spiked in pH 7 at 4°C in triplicates are shown. Due to limitations in the set-up of the pH experiment, the last four time points (100, 123, 146 and 173 h) are only measured in duplicates. The percentage of the area ratios of 4HDPA (left side) and of QMI (right side) on the sum of both species is plotted via the time (h). Error bars indicate confidence intervals of 95%. The red line indicates the linear regression in the form of y = a + b*x. Values are given in the figure.

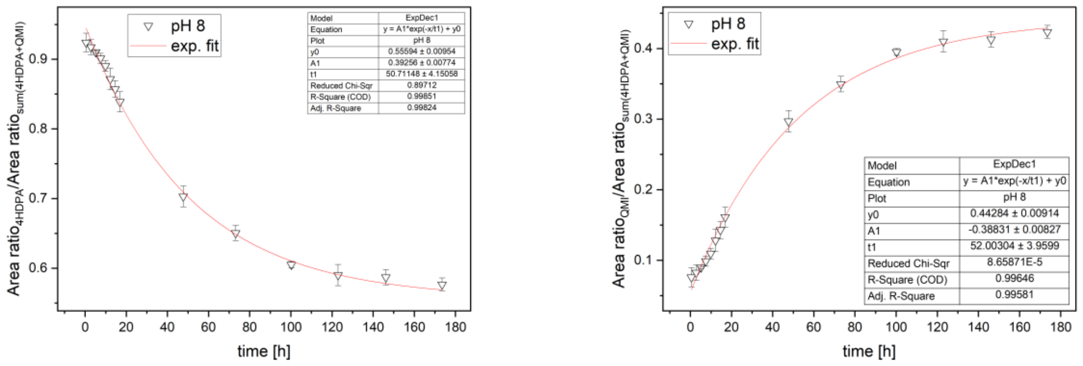

Figure S13: The results of the pH stability experiments of 4HDPA (c = 50 µg/L) spiked in pH 8 at 4°C in triplicates are shown. Due to limitations in the set-up of the pH experiment, the last four time points (100, 123, 146 and 173 h) are only measured in duplicates. The percentage of the area ratios of 4HDPA (left side) and of QMI (right side) on the sum of both species is plotted via the time (h). Error bars indicate confidence intervals of 95%. The red line indicates an exponential regression in the form of y = A1*exp(-x/t1) + y0. Values are given in the figure.

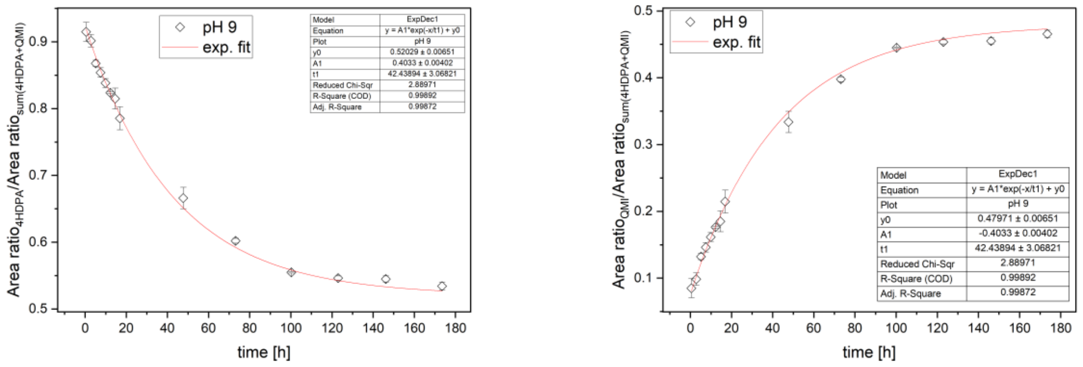

Figure S14: The results of the pH stability experiments of 4HDPA (c = 50 µg/L) spiked in pH 9 at 4°C in triplicates are shown. Due to limitations in the set-up of the pH experiment, the last four time points (100, 123, 146 and 173 h) are only measured in duplicates. The percentage of the area ratios of 4HDPA (left side) and of QMI (right side) on the sum of both species is plotted via the time (h). Error bars indicate confidence intervals of 95%. The red line indicates an exponential regression in the form of y = A1*exp(-x/t1) + y0. Values are given in the figure.

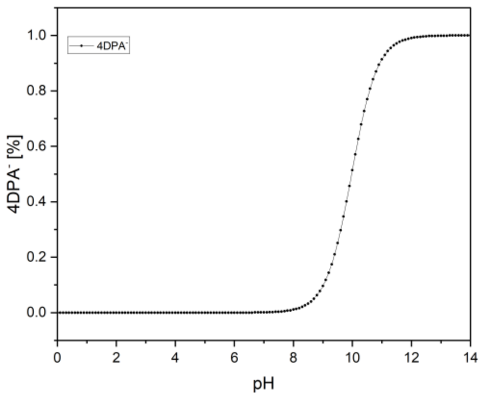

Figure S15: Distribution in percent of the deprotonated anion $4DPA^-$ depending on pH and the pK_a value of 4HDPA is 9.97, both calculated using Marvin[7].

5.3 Ingredients of YES exposure medium

Table S7: Ingredients of the exposure medium in the yeast estrogen screen (YES) and the planar p-YES according to ISO 19040-1:2018(E)[8], Riegraf et al.[9] and Schoenborn et al.[10]. The yeast nitrogen base without amino acids was from Sigma Aldrich.

solution	component	Single component				YES medium		p-YES medium	
		**m** [g]	**V** [L]	**conc.** [g/L]		**V** [mL]	**conc.** [g/L]	**V** [mL]	**conc.** [g/L]
10xSD-Medium	yeast nitrogen base without amino acid	67	1	67		4.2	27.5	4.2	27.9
	glucose	200		200			82.1		83.2
10xMcD-Do-Medium	L-lysine-HCl	0.175	0.5	0.35		4.2	0.1	4.2	0.1
	L-histidine-HCl	0.12		0.24			0.1		0.1
Glucose solution	glucose	144	0.5	288		1.6	45.0	1.6	45.6
$CuSO_4$ solution	$CuSO_4 * 5\ H_2O$	0.25	1	0.25		0.099	0.0024	0.098	0.0024
Ampicillin solution	ampicillin sodium salt	1	0.01	100		0.067	0.7	–	–
Streptomycin solution	streptomycin sulfate salt	1	0.01	100		0.067	0.7	–	–
					sum	10.23		10.10	

6. Quantification of estrogenic potential

6.1 Effect data of the Yeast Estrogen Screens

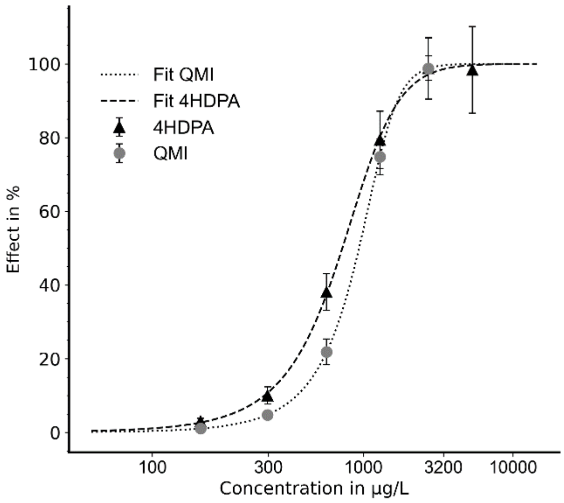

Figure S16: Concentration-response curves and raw data (n = 3) with referring 95% confidence intervals of 4HDPA and QMI (n = 4) are shown. Y-axis shows percentage of effect, x-axis shows concentration in µg/L on a logarithmic scale. EC-values can be seen in Table S10.

Table S8: Underlying Data for the calculation of concentration-response relationships of 4HDPA in the YES. Three replicates and their mean and 95 % confidence intervals (CI) are shown.

**Concentration** [mg/L]	**Replicate 1** [corrected AU]	**Replicate 2** [corrected AU]	**Replicate 3** [corrected AU]	**Mean** [corrected AU]	**95% CI**
5	17	17	13	16	2.2
2.5	16	17	16	16	0.61
1.25	11	13	14	13	1.5
0.63	6.0	6.0	7.8	6.6	0.94
0.31	1.8	2.2	2.8	2.3	0.47
0.16	1.0	1.2	1.4	1.2	0.18

Table S9: Underlying Data for the calculation of concentration -response relationships of QMI in the YES. Four replicates, which are divided in eight technical replicates, their mean and 95 % confidence intervals (CI) are shown.

**Concen-tration** [mg/L]	**Replicate 1.1** [corrected AU]	**Replicate 2.1** [corrected AU]	**Replicate 3.1** [corrected AU]	**Replicate 4.1** [corrected AU]	**Mean** [corrected AU]	**95% CI**
2.5	12	16	13	13	13	1
1.25	9.9	11	9.4	9.3	9.8	0.6
0.63	3.8	4.4	2.5	2.9	3.3	0.5
0.31	1.9	1.3	0.83	0.92	1.2	0.3
0.16	1.3	0.75	0.44	0.53	0.76	0.25
0.08	1.4	0.55	0.35	0.38	0.65	0.28
**Concen-tration** [mg/L]	**Replicate 1.2** [corrected AU]	**Replicate 2.2** [corrected AU]	**Replicate 3.2** [corrected AU]	**Replicate 4.2** [corrected AU]		
2.5	13	12	10	11		
1.25	11	10	8.3	8.9		
0.63	4.2	3.5	2.5	2.5		
0.31	1.7	1.3	0.81	0.87		
0.16	1.4	0.70	0.47	0.49		
0.08	1.3	0.57	0.33	0.35		

Table S10: Estimated effect concentrations, using a five parametric function, and corresponding 95%-confidence intervals (CI) for 4HDPA and QMI. Values are based on the means of each replicate estimated separately.

	**4HDPA**		**QMI**	
**Effect Level** [%]	**Effect Concentration** [µg/L]	**95% CI**	**Effect Concentration** [µg/L]	**95% CI**
10	330	59	440	21
20	460	69	600	26
30	580	89	740	30
40	690	120	850	33
50	820	180	960	36
60	980	260	1100	43
70	1200	410	1200	60
80	1500	700	1400	100
90	2300	1500	1600	200

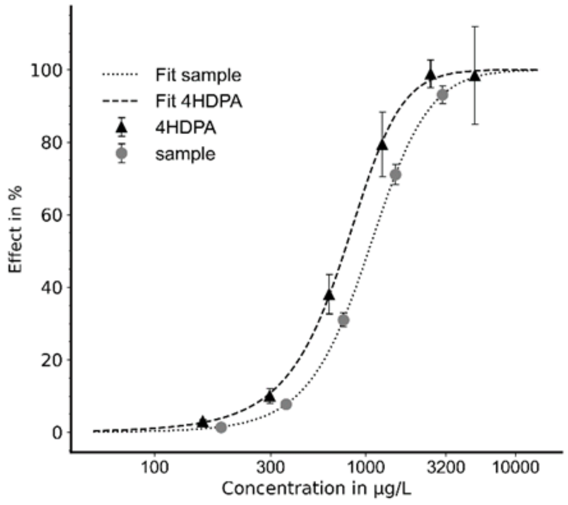

Figure S17: Concentration-response curves and raw data (n = 3) with referring 95% confidence intervals of 4HDPA and the leachate are shown. Y-axis shows percentage of effect, x-axis shows concentration in µg/L on a logarithmic scale. On the basis of the toxicological data, BEQs and RPs were calculated (ISO 23169; ISO, 2022) to determine the percentage estrogenic effect of 4HDPA on the whole sample.

7. NTS-portal search

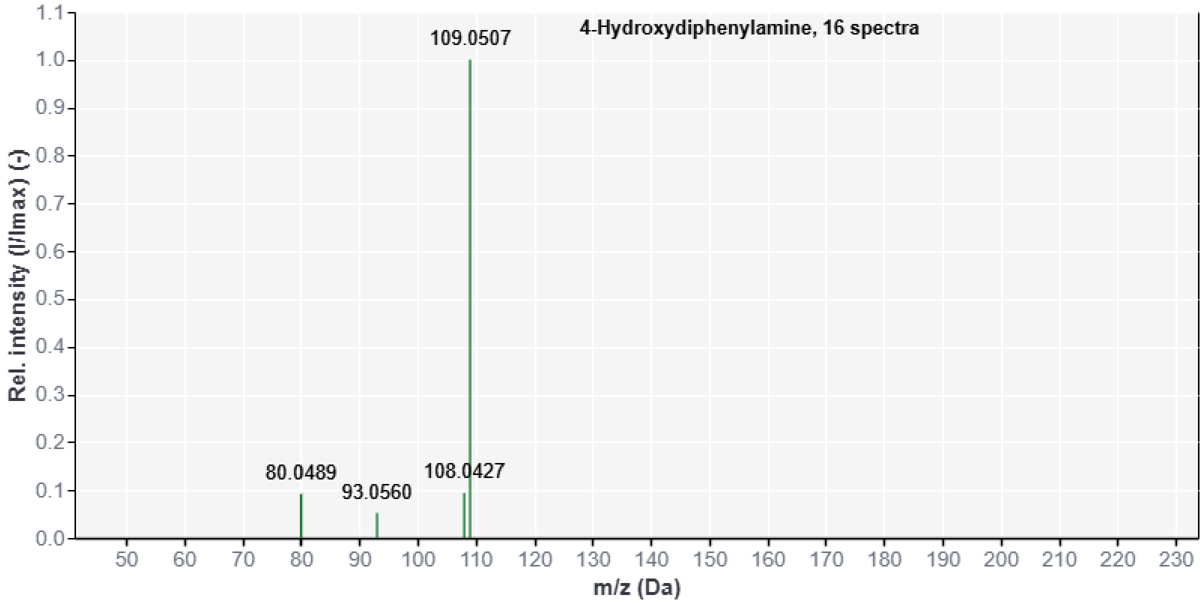

Figure S18: Average MS^2 spectrum of 4HDPA in the NTS-portal[11,12].

Table S11: Data table exported from the NTS-portal[11,12] with the compound 4-hydroxydiphenylamine (4HDPA) detected in water. Overview over the date of sampling, station and relative peak area (RPA) in relation to the internal standard bezafibrate-d4 which is added to each sample before analysis for quality control. The concentrations were determined using a semi-quantification approach (SQ).

date	Measurement station		Compound	RPA	Conc. (SQ) [ng/L]	Matrices
05.07.2019	rhein_ko_l		4HDPA	0.003	7.6	water
06.07.2019	rhein_ko_l		4HDPA	0.017	14	water
07.07.2019	rhein_ko_l		4HDPA	0.015	13	water
08.07.2019	rhein_ko_l		4HDPA	0.013	12	water
19.07.2019	rhein_ko_l		4HDPA	0.001	6.7	water
21.07.2019	rhein_ko_l		4HDPA	0.001	6.7	water
23.07.2019	rhein_ko_l		4HDPA	0.001	6.7	water
24.07.2019	rhein_ko_l		4HDPA	0.003	7.6	water
25.07.2019	rhein_ko_l		4HDPA	0.002	7.1	water
26.07.2019	rhein_ko_l		4HDPA	0.005	8.5	water
27.07.2019	rhein_ko_l		4HDPA	0.008	9.8	water
28.07.2019	rhein_ko_l		4HDPA	0.004	8.0	water
29.07.2019	rhein_ko_l		4HDPA	0.002	7.1	water
30.07.2019	rhein_ko_l		4HDPA	0.016	13	water
05.07.2021	parthe_1.45		4HDPA	0.002	7.1	water
07.07.2021	vereinigte_weisseritz_1.2		4HDPA	0.018	14	water
07.07.2021	oelsabach_15		4HDPA	0.006	8.9	water
		**pH**				
03.05.2024	nordumfluter_rubber-dam_1	8.2	4HDPA	0.035	22	water
03.05.2024	nordumfluter_rubber-dam_2	8.0	4HDPA	0.030	20	water
03.05.2024	nordumfluter_rubber-dam_3	7.9	4HDPA	0.027	18	water

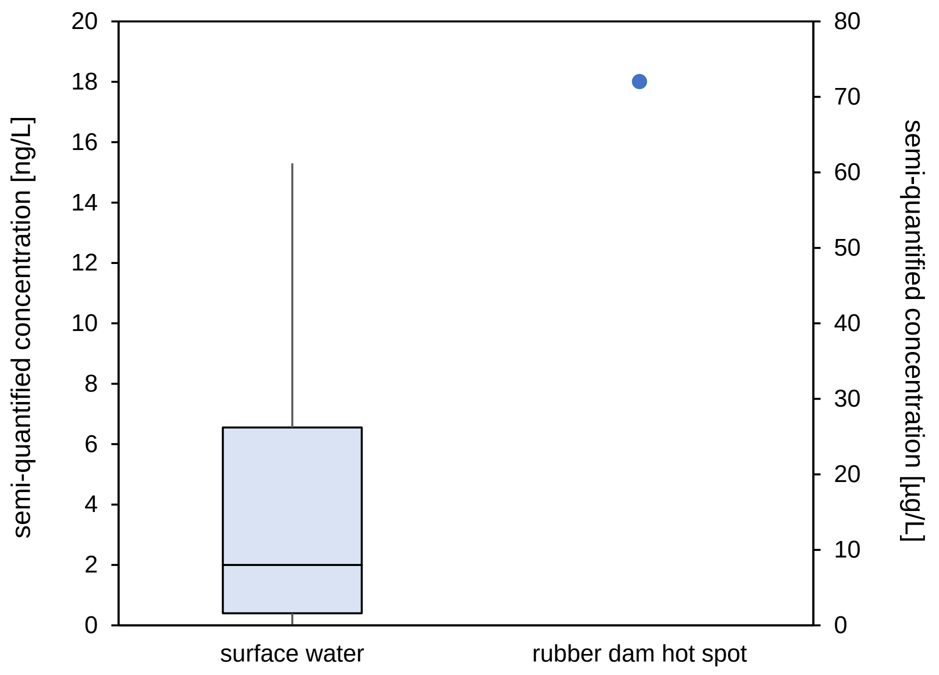

Figure S19: Semi-quantified concentrations of 4HDPA relatively to bezafibrate-d4 which was added to each sample as internal standard for nomination and quality control. Results are shown for the German rivers in which the 4HDPA was detected in the years 2019, 2021 and 2024 in the NTS portal[11,12] (left side) and one hot spot where a rubber dam was installed (right side). All concentrations were evaluated in retrospective.

8. References

(1.) Baragona, F.; Lomberget, T.; Duchamp, C.; Henriques, N.; Lo Piccolo, E.; Diana, P.; Montalbano, A. and Barret, R. Synthesis of 5-substituted 2,3-dihydrobenzofurans in a one-pot oxidation/cyclization reaction*. Tetrahedron*. **2011**, 67(45), p. 8731-8739. DOI: 10.1016/j.tet.2011.09.020.

(2.) Fetizon, M.; Balogh, V. and Golfier, M. Oxidations with silver carbonate/celite. V. Oxidations of phenols and related compounds*. J. Org. Chem. .* **1971**, 36(10), p. 1339–1341. DOI: 10.1021/jo00809a004.

(3.) Adesina, A.D. **2020**. *Molecular Diversity from N-phenylquinoneimine.* (Doctor of Philosophy). Newcastle University,

(4.) Dierkes, G.; Lauschke, T.; Becher, S.; Schumacher, H.; Foldi, C. and Ternes, T. Quantification of microplastics in environmental samples via pressurized liquid extraction and pyrolysis-gas chromatography*. Anal Bioanal Chem*. **2019**, 411(26), p. 6959-6968. DOI: 10.1007/s00216-019-02066-9.

(5.) DIN, Chemische Analytik - Verfahren der Standardaddition - Verfahren, Auswertung. **1998**, DIN. p. 7.

(6.) Jewell, K.S.; Kunkel, U.; Ehlig, B.; Thron, F.; Schlusener, M.; Dietrich, C.; Wick, A. and Ternes, T.A. Comparing mass, retention time and tandem mass spectra as criteria for the automated screening of small molecules in aqueous environmental samples analyzed by liquid chromatography/quadrupole time-of-flight tandem mass spectrometry*. Rapid Commun. Mass Spectrom. .* **2020**, 34(1), p. e8541. DOI: 10.1002/rcm.8541.

(7.) Marvin. https://www.chemaxon.com. (accessed 2025-05-01).

(8.) ISO, Water quality -Determination of the estrogenic potential of water and waste water - Part 1: Yeast estrogen screen (Saccharomyces cerevisiae). **2018**, International Organization for Standardization.

(9.) Riegraf, C.; Reifferscheid, G.; Moscovici, L.; Shakibai, D.; Hollert, H.; Belkin, S. and Buchinger, S. Coupling high-performance thin-layer chromatography with a battery of cell-based assays reveals bioactive components in wastewater and landfill leachates. *Ecotoxicol. Environ. Saf.* . **2021**, 214. DOI: 10.1016/j.ecoenv.2021.112092.

(10.) Schoenborn, A.; Schmid, P.; Bram, S.; Reifferscheid, G.; Ohlig, M. and Buchinger, S. Unprecedented sensitivity of the planar yeast estrogen screen by using a spray-on technology. *J. Chromatogr., A*. **2017**, 1530, p. 185-191. DOI: 10.1016/j.chroma.2017.11.009.

(11.) Jewell, K.S.; Lessmann, O.; Thron, F.; Skottnik, J.; Tuchscherer, I.; Wick, A. and Ternes, T.A. **2024**. ntsportal: A Non-Target Screening Data Archive and Distribution Tool (Version R package version 0.1.0). https://github.com/bafg-bund/ntsportal. (accessed 2025-08-14).

(12.) Jewell, K.S.; Dietrich, C.; Köppe, T.; Thron, F.; Wick, A. and Ternes, T.A. **2023**. ntsworkflow: A Non-Target Screening Data Evaluation Tool (Version R package version 0.2.2). https://github.com/bafg-bund/ntsworkflow. (accessed 2025-08-14).
